# Effects of signalling tax evasion on redistribution and voting preferences: Evidence from the Panama Papers

**DOI:** 10.1371/journal.pone.0229394

**Published:** 2020-03-10

**Authors:** Laila Ait Bihi Ouali

**Affiliations:** Dept. of Civil and Environmental Engineering, Imperial College London, London, United Kingdom; TED University, TURKEY

## Abstract

This paper provides empirical evidence that individuals substantially revise their stated wealth redistribution preferences after fiscal scandals. The 2016 Panama Papers scandal revealed top-income tax evasion behaviour simultaneously worldwide. The empirical investigation exploits this event as a quasi-natural experiment. I rely on two original datasets, a UK household longitudinal dataset and a survey conducted in 22 European countries. I use a difference-in-differences strategy and find that pro-redistribution statements increased between 2% and 3.3% after the scandal. Responses are heterogeneous and larger for right-wing individuals and low-income individuals. This change in wealth redistribution preferences is likely to have been translated into a slight change in votes. The results suggest an increase in stated voting intentions for the left and a decrease for the right. Complementary estimations reveal that more media coverage and more individuals involved by country increase the magnitude of the response.

## Introduction

Do people revise their views of inequality and taxation after an informational shock? In the past decade, leaks have uncovered tax evasion behaviours, successively exposing firms and top-income earners both at the country level and worldwide. Tax avoidance leads to an approximate $600 billion annual tax loss, divided roughly into $400 billion in OECD countries and $200 billion elsewhere [1]. A recent and substantial strand of literature investigates the extent of tax evasion with the aim to quantify it. This literature namely shows that top-income tax evasion is substantial and occurs especially at the very top of the income distribution [2]. Using information from the Panama Papers, they find that, on average, 3% of personal taxes are evaded while between 25% and 30% are evaded by the top 0.01%. Such information could trigger an increase of inequality beliefs.

Tax havens generate inequality, which in turn leads to the question of the optimality of taxation systems. The possibility to resort to offshore firms to avoid paying taxes is not included in the design of optimal taxation policies. This provides a motivation to test whether individuals are sensitive to the existence of inequality and update their stated preferences accordingly. If so, this would advocate for inclusion of these behaviours in the design of optimal policies.

To the best of my knowledge, this study is the first to provide an estimation of the Panama Papers scandal impact on individual preferences. This paper uses this fiscal scandal as a quasi-natural experiment. The Panama Papers generate a time discontinuity: it was leaked worldwide on April 3, 2016, in various media. Information on potential tax evasion leaked from a source working at Mossack Fonseca, a law firm in Panama involved with offshore companies: the motivations of the anonymous source (named “John Doe”) were ethical and exogenous. Therefore, the leak was unanticipated by individuals.

The Panama Papers leaked names of people involved in offshore companies. Given that using offshore companies is not necessarily illegal, the Panama Papers do not provide clear proof of illegal activity. Yet offshore companies continue to be a means to avoid taxes. Therefore, the Panama Papers are a signal of top-income tax evasion: this scandal showcases the ease with which wealth can be concealed in tax havens ($32 trillion offshore, according to the Tax Justice Network (for more details, see [3]). The important media coverage also shed a negative light on these practices. Therefore, the informational shock marks the Panama Papers as a signal of top-income tax evasion.

Prior research has tested the impact of informational shocks on individuals [4–7]. Negative experiences are found to change individuals’ preferences and make them less optimistic or more risk averse [8, 9]. Fiscal scandals are considered a negative experience, triggering a revision of beliefs. Studies testing firms’ reactions to the 2008 Liechtenstein tax affair and the Panama Papers (respectively [10, 11]) find that leaks increase tax haven withdrawals and decrease the market value of firms involved in the scandal. More precisely, the Panama Papers erased $135 billion in market capitalization among 397 public firms [11].

I use panel data from the British Election Study (BES) for the years 2014-2016. This dataset follows the same individuals in the United Kingdom and contains indicators of media exposure. I resort to a difference-in-differences methodology in which the treated individuals are informed on politics and current affairs via various media: television, radio, newspapers, and Internet. Mass media is a major source of information for the general public and is the motivation behind this treatment. Both theoretical and empirical studies find that information encourages individuals to update their beliefs on the matters of taxation and wealth redistribution. In addition, incomplete or biased information affects redistribution preferences in countries with high inequality levels [12].

This study focuses on both wealth redistribution and voting outcomes in the form of stated voting intentions. The results show that preferences for top-income redistribution strongly and abruptly increase after the Panama Papers scandal. I find that the probability to ‘strongly agree’ with redistribution statements increases by 15 percentage points after the scandal. Preferences for redistribution are greater when individuals have a strong preference for low inequality but observe greater inequality in their country, which is in line with the corresponding literature [13]. By exposing tax avoiders, the Panama Papers highlight a source of inequality, which triggers the revision of beliefs. Falsification tests indicate that this change in preferences is driven not only by the political context and the perception of the government but also through the redistribution channel. I find no differentiated effect based on socio-demographic variables (*e.g*., age, gender). However, I observe heterogeneity based on the household income level, with a larger effect on households earning between £10,000 and £39,999 per year. I observe heterogeneous responses with regard to political affiliation. The propensity to update beliefs is higher for right-wing individuals; conversely, I do not observe a heterogeneous effect for left-wing individuals, which can be attributed to a ‘ceiling’ effect [9] as they already state higher preferences for wealth redistribution.

In addition, I test whether the scandal affects voting outcomes, namely stated voting intentions. Relying on the median-voter model, I expect a larger gap between average and median income to lead to an increase in preferences for redistribution [14]. After the scandal, I find an increase in stated voting intentions for the left and the centre but no effects for the right. Therefore, this scandal appears to trigger polarisation; yet, I find a decrease in the certainty of stated voting intention for right-wing parties. This shows that the scandal triggered instability in stated voting intentions. Thus, fiscal scandals encourage individuals to take a stand and strongly prefer wealth redistribution.

I use the European Social Survey (ESS) to obtain information at the European level and to complement the analysis for the years 2014 to 2016 at the European level using the same difference-in-differences methodology. I find stable results on preferences for redistribution, which verifies that post-scandal outcomes are similar across Europe. Additional estimations at the European level indicate that the increase of pro-redistribution statements is positively correlated with the media coverage intensity. The Panama Papers involved individuals from different nationalities and from almost all European countries. It is interesting to test whether individual reactions differ in countries where at least one fellow citizen is involved in the scandal. I test for a differentiated response with respect to the intensity of the scandal (*i.e*., media coverage and the number of locals involved) and find that the increase of pro-redistribution statements grows with the intensity of the scandal in a country.

This paper contributes to the literature by providing a quantification of the elasticity of redistribution preferences to the provision of information. It checks whether the change in the perception of inequality triggers a change in stated voting intentions. This study uses the Panama Papers event as a quasi-natural experiment, given that the informational shock was unanticipated and exogenous. This scandal provides evidence of the existence of top-income tax evasion in real life. In turn, this ensures higher external validity of my findings and complements randomized controlled experiments relying on Internet survey responses.

The framework in this paper aims to provide a more effective identification strategy and broader external validity. The drivers of redistribution preferences are the subject of a stream of literature that often uses survey data to link individual traits to preferences [8, 13, 15, 16]: in these studies, respondents simply answer non-experimental survey questions about their views on policy and social preferences. The difference-in-differences methodology in this paper disentangles the impact of the shock and identifies causal effects instead of correlations. A more recent strand of literature resorts to randomized online survey experiments to attain updates in preferences after an informational intake. Studies find that informational shocks influence individuals’ stated views on inequality [5, 6]. Although experiments increase the quality of the identification strategy compared with simple survey studies, they are undermined by limited external validity. In addition, randomized controlled experiments that use Amazon Mechanical Turk [6] include participants who have a financial incentive to provide the expected responses, as this may affect their rating on the platform and, therefore, their future earnings. The current study provides a real-life experiment that corroborates previous research findings and provides external validity.

The rest of this paper is organised as follows: Section 2 introduces and explains the tax evasion mechanism uncovered by the Panama Papers scandal. Section 3 presents the data along with the empirical strategy. Section 4 shows that the Panama Papers leak increased individuals’ wealth redistribution preferences and altered voting outcomes; it also provides robustness checks. Section 5 presents complementary estimations and corroborates the effects of the scandal at the European level. Section 6 concludes.

## The Panama Papers scandal

### 2.1 An exogenous leak

The Panama Papers scandal began with a leak from an anonymous source working in a law firm in Panama called Mossack Fonseca. This source contacted a German investigative reporter at the *Süddeutsche Zeitung* through an encrypted messaging service. The motivations of this whistleblower (named ‘John Doe’) were exogenous, as his stated motivation was to “make these crimes public”. Excerpts of the conversation are available on the ICIJ’ Panama Papers website . The documents were first examined by a consortium of journalists, called the International Consortium of Investigative Journalists (ICIJ), which is also the origin of other leaks, including the Swiss Leaks and the Offshore Leaks. It is important to note that the magnitude of the Panama Papers leak is larger than any of the previous leaks uncovered by the ICIJ: the anonymous source shared more than 11.5 million documents on 241,488 companies in which 14,153 individuals were involved. Data cover a large period (1977–2015). The exogenous nature of this leak and the fact that it was unanticipated by the public motivate its use as a quasi-natural experiment.

### 2.2 A signal of tax evasion

The Panama Papers do not constitute clear proof of illegal activity. These papers essentially leaked names of people involved in offshore companies. The use of offshore companies is not necessarily illegal; however, these companies constitute a means to avoid paying taxes. The mechanism behind offshore companies is rather simple and can be summarized in two steps. To create a shell company, individuals choose a tax haven and set a corporation there. They hire a nominee responsible to run their business. Then they open a bank account, either in that region or in another tax haven and then move money into their account to access their money. The first step is to create a “shell company” registered in a tax haven. The shell company is run by a nominee so that the name of the avoider does not directly appear on any related documents. The second step is to open a bank account in the same tax haven and wire money from the corporation to the bank account. However, this mechanism can be used to launder money.

Therefore, offshore companies can be a signal of top-income tax evasion: the Panama Papers scandal highlights how easy it is to conceal wealth in tax havens. The media coverage of the Panama Papers was intensive and very negative: S1 and S2 Figs show the high press coverage of the scandal by newspapers in the UK in 2016. S4 Table shows that even right-wing newspapers condemned the top-income earners mentioned in the Panama Papers. Therefore, the informational shock of the Panama Papers scandal presents a signal of top-income tax evasion.

### 2.3 Magnitude of the scandal

Although the information initially came through to a German reporter, the ICIJ reported this information in various media worldwide. S1 Table provides the list of the reporting partners of the ICIJ in both Europe and the US, encompassing numerous sources with a wide audience.

**Fig 1:**
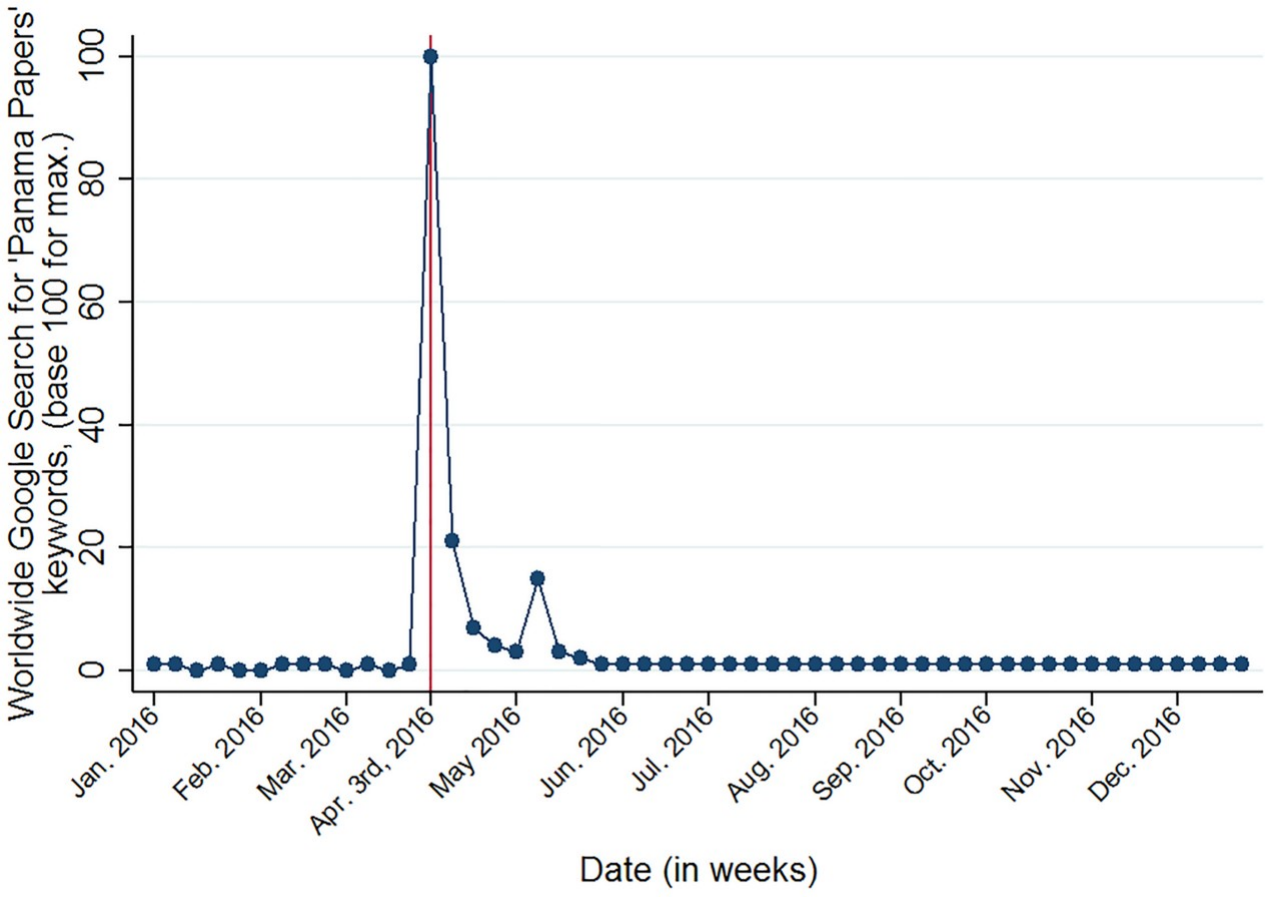
Internet search intensities worldwide for keyword “Panama Papers” in 2016 by volume (Source: Google trends).

It is crucial to document the intensity and extent of the media exposure, as most individuals likely found out about the Panama Papers through these sources. Fig 1 presents the evolution of the web search intensity for the keyword “Panama Papers” and shows a sudden increase in search intensity from April 3, 2016, onward. Using time discontinuity based on an informational leak from various media worldwide is thus a sound strategy. This proves that the leak expanded worldwide, thus further motivating the European analysis of the variation in individuals’ responses after the scandal. Fig 1 shows a large increase in web searches for the keyword “Panama Papers”, with a second surge in May 2016. This is probably due to the intense political debates (*i.e*., the Brexit campaign, as presented in the timeline, see S3 Fig). However, falsification tests show that redistribution preference is the main channel.

## Empirical strategy and data

### Data

#### UK and European data

Data used in this paper come from two sources: longitudinal UK data and European survey data. This section presents the characteristics of both datasets. Longitudinal survey data come from the BES (for detailed information on the source, see [17]). from 2015 to 2016 and constitute a sample of 305,912 observations covering 53,388 respondents. The data contain detailed questions on personal redistribution preferences and interview dates. I use the latter to define exposure to the Panama Papers scandal (**i.e**., responses after April 3, 2016). The data provide enough counterfactuals through recurrent waves. The BES respondents are interviewed up to four times in the sample of interest; on average, they are interviewed twice. Use of the same individuals constitutes a means to record any personal shifts by controlling for unobserved characteristics in the empirical analysis. S5 Table presents summary statistics and a comparison with census data, suggesting that the BES is representative of the population.

The BES dataset provides two types of information relevant for this study: information habits and redistribution preferences. First, respondents state how much time they spend following politics and current affairs in a standard week via four possible media types: (i) television, (ii) radio, (iii) newspapers, (iv) Internet and (v) through people they know. The last item corresponds to people being asked about how much time they spend following politics and current affairs through individuals they know telling them about the news. This set of information is crucial to define the treated (informed) and control (uninformed) groups. We use this information to build a dummy variable equal to 1 if the respondents say they spend time getting information through one of the five media channels and 0 otherwise. This dummy variable defines the treatment.

Second, the BES questionnaire also contains relevant information on redistribution preferences. Respondents face a series of statements on which they are asked how much they agree or disagree with them on a 5-point scale (1 = completely disagree, 5 = completely agree). The statements used in this paper are as follows: “Government should redistribute income from the better to the worse off”, “Government should try to make income more equal”, and “Ordinary people do not get their fair share of the nation’s wealth”.

The second database used in this analysis is the ESS, which records information for 22 European countries, for the years 2015 and 2016. The final sample contains 100,322 observations. The ESS is a cross-sectional dataset, implying that this sample contains information on 100,322 individuals. Similar to the BES, this dataset contains detailed questions on personal redistribution preferences and interview dates.

#### Descriptive statistics

This section presents descriptive statistics that anticipate and illustrate the main findings. S6 Table shows that the socio-demographic characteristics of both the treated and control groups are rather similar. Informed individuals’ characteristics are more similar to all respondents than are the uninformed individuals.

**Fig 2:**
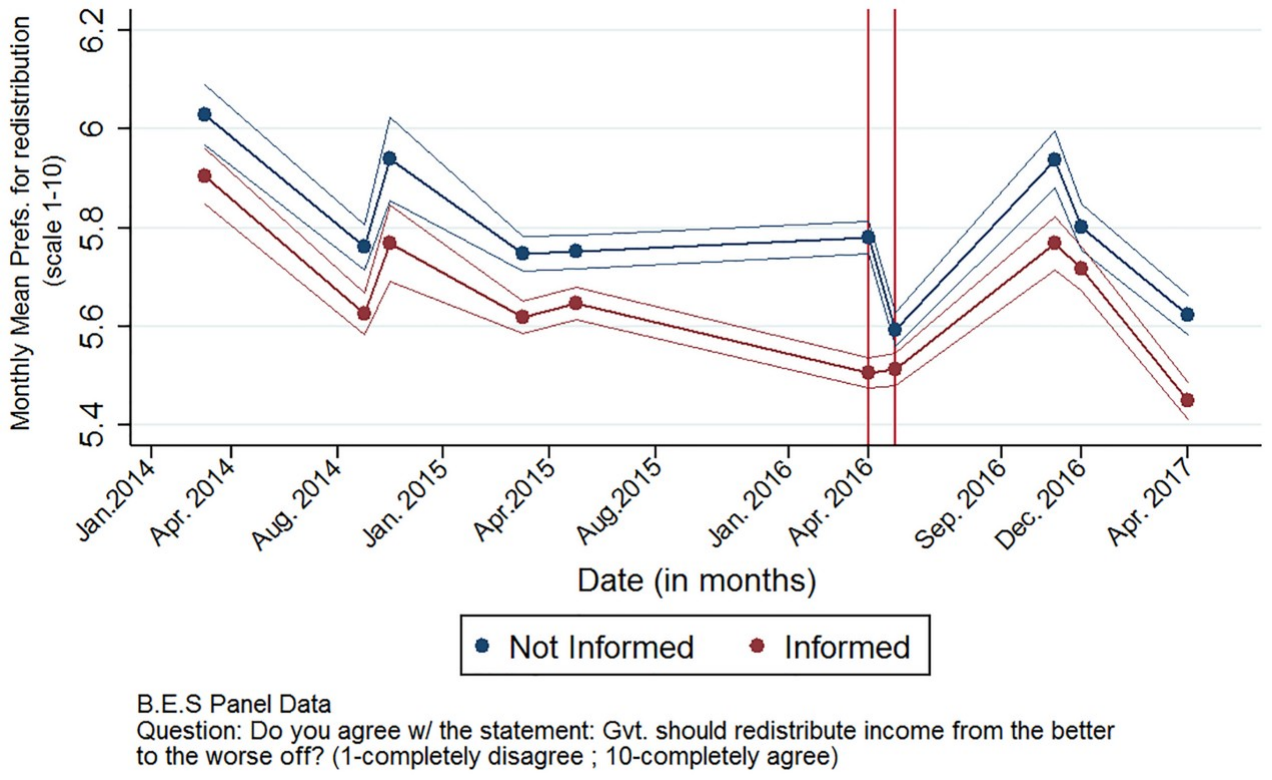
Evolution of stated redistribution preferences over time.

Fig 2 provides graphical proof of the common trend between the ‘informed’ (treated) and ‘uninformed’ (control) groups. It shows a clear parallel trend between the two groups in their preferences for wealth redistribution over time until the Panama Papers leak. Then, when the scandal happens in April 2016, the difference between the groups becomes significantly larger. Therefore, the common trend is clear, and post-leak variations do not present overlapping areas. The only break in the common trend (i.e., difference between control and treated groups) occurs right after the scandal broke in April 2016.

The distribution of the treated and control groups is as follows: the sample of informed individuals contain 274,016 observations for 47,580 individuals (i.e., 89% of the sample) while we have 31,896 observations for 5,808 individuals (i.e., 11% of the sample). This difference in shares of individuals between the treated and untreated groups does not impair the quality of this analysis, as all criteria that matter when using a difference-in-differences strategy alter trends. Fig 2 shows that before the informational shock, the common pre-treatment trend between the two groups is maintained. After the scandal breaks in the media, the responses of the treated and control groups diverge, which hints at the fact that the Panama Papers had a differentiated response on each group. Yet, the response of individuals to that informational shock appears to be limited over time as a few months after, individuals’ responses appear to go back to following a common trend. The empirical strategy used in this study yields results that are robust to various specifications.

### Empirical model and identification

This paper uses a difference-in-differences methodology. This strategy includes the exact date of the leak worldwide (April 3, 2016) as a time discontinuity. The main identifying assumption is that, conditional on both the vector of socio-economic characteristics and time trends and on individual unobserved heterogeneity, the interview date is exogenous to the Panama Papers scandal. In 2016, roughly half of all respondents in this sample completed interviews on each side of the cut-off date —that is, before and after April 3. Datasets provided at the time of this study with relevant variables do not have a consistent inflow of individuals around the cut-off date, which prevents the possibility to use a regression discontinuity framework.

The control group consists of all uninformed individuals. More precisely, the BES contains questions on the intensity (time) with which respondents follow politics and current affairs from a set of sources: (i) media (television, radio, newspapers, and Internet) and (ii) people. Uninformed individuals (control) are those who spend no time getting news through any of these channels. Informed individuals include all individuals informed before and after the scandal through at least one of these channels.

All dependent variables in this study share a common structure. They question whether individuals agree or disagree with a given statement on a 5-point (completely disagree/completely agree) scale. An exception is the statement “Government should redistribute income from the better to the worse off”, which is rated on a 10-point scale. I use the following specification for all outcomes of interest:

$$\begin{array}{c} y_{it}=\beta_{0}+\sum_{k=1}^{n}\beta_{k}x_{it}^{k}+\delta_{1}Post_{it}+\delta_{2}Informed_{it}+\delta_{3}Post_{it}*Informed_{it}+\phi_{i}+\epsilon_{it} \end{array}$$ (1)

where *y*_*it*_ defines the dependent variable, $x_{i}^{k}$ is the vector of individual observable characteristics and time controls, and *ϵ*_*it*_ is the error term. *Informed*_*i*_ is a dummy variable that equals 1 if individuals are informed by television, radio, newspapers, Internet, or other individuals and 0 otherwise. The time window that is covered by the post variable is the same as the overall data window and finishes on 31 December 2016. *Post*_*i*_ is a dummy variable that equals 1 if the interview occurs after April 3, 2016, and 0 otherwise. Moreover, *ϕ*_*i*_ represent individual fixed effects (in panel fixed effects regressions only), and *δ*_3_ is the average treatment effect on the treated group. Following previous findings in the literature, I expect an increase of preferences for redistribution for treated individuals after the leak (i.e., *δ*_3_ > 0). Given that all variables of interest are ordinal variables with five cases, I use ordered probit specifications for all regressions.

## Results

### Baseline results

This section presents and discusses the effects of the Panama Papers on preferences for redistribution. This study uses a difference-in-differences methodology. Estimates from our main specification, ordered probit, are presented in Table 1. Complementary estimations use OLS and panel methods respectively in S9 and S10 Tables.

**Table 1:** Effect of the Panama Papers scandal on preferences: Ordered probit estimates.

	Government should…		Ordinary people do not get their fair share of nation’s wealth
	…redist. from better to worse off	…try to make income more equal	
	(1)	(2)	(3)
Post Apr. 3rd, 2016 (Yes = 1)	-0.135***	-0.110***	-0.142***
	(0.0258)	(0.0340)	(0.0269)
Informed (Yes = 1)	-0.0276*	-0.122***	0.0455**
	(0.0148)	(0.0180)	(0.0180)
**Post Apr. 3rd, 2016 & Informed (Yes = 1)**	**0.0581** **	**0.0952** ***	**0.0881** ***
	**(0.0241)**	**(0.0274)**	**(0.0283)**
Time Controls	Yes	Yes	Yes
Controls socio dem	Yes	Yes	Yes
Control income	Yes	Yes	Yes
Observations	63,293	94,035	63,921

Standard errors are in parentheses and clustered at the interview date level.

*** p<0.01,

** p<0.05,

* p<0.1

Source: BES W13 Panel v1.2, own calculations.

#### Socio-demographics and economic variables

S8 Table presents estimates of the impact of socio-demographics and economic variables. The table shows that socio-demographic characteristics influence the propensity for redistribution. Older people are significantly more likely to agree with pro-redistribution statements than younger people. Political affiliation influences preferences, a finding in contrast with that of [6], who find mild to no effects. I find that support of more redistributive policies decreases for larger household income levels, which is in line with prior research findings [18].

S8 Table also captures the effect by occupation. Retired people have lower redistribution preferences, and the unemployed have higher preferences than full-time workers. These results corroborate the literature which shows that the personal experience of economic hardship, particularly the loss of a job, has a strong effect on increasing support for welfare spending. [9]. The results also corroborate an increase in preferences of the unemployed and individuals with lower wages. Finally, respondents with more trust in the government have lower preferences with redistribution. This is in line with the literature [6] which finds that individuals who perceive less inequalities tend to show a larger trust in the government. Therefore, S8 Table results are comparable to the findings in the literature.

#### Effects of the scandal on preferences for redistribution

The results, summarized in Table 1, show that the Panama Papers scandal triggered a response at the extensive margin. First, across all specifications, I consistently find that respondents’ agreement with the item “Ordinary people do not get their fair share of the nation’s wealth” increased after the Panama Papers event. I also find that individuals are more likely to agree with the statements “Government should try to make income more equal” and “Government should redistribute income from the better to the worse off” after the scandal. This indicates a positive impact of the informational shock on the perception of inequality. It also corroborates findings that informed individuals increase their stated redistribution preferences after a scandal [6]. In their experiment, respondents preferred a higher tax rate on the top 1% of income, significantly encouraged the scope of government activity and were more in favour of an increase of the minimum wage. Therefore, the quasi-natural experiment validates laboratory experiment findings. The results are stable to both panel fixed effects and ordinary least squares (OLS) specifications presented in S9 and S10 Tables.

#### Marginal effects

I use estimates from all ordered probit regressions to determine the marginal responses for all the dependent variables. The most natural way to interpret ordered response models (and discrete probability models in general) is to determine how a marginal change in one regressor changes the distribution of the outcome variable, *i.e*. all the outcome probabilities. The main focus in the analysis of ordered data should be put on the conditional cell probabilities given by

$$\begin{array}{c} Pr\left[y=j|x\right]=F\left(\mu_{j}-x^{\prime }\beta \right)-F\left(\mu_{j-1}-x^{\prime }\beta \right). \end{array}$$ (2)

with *F* being the variance of the distribution function and *β* the vector of coefficient attached to the vector of observable variables *x*. In order to identify the parameters of the model we have to fix location and scale of the argument in *F*, the former by assuming that *x* does not contain a constant term, the latter by normalizing the variance of the distribution function *F*

$$\begin{array}{c} MPE_{jl}\left(x\right)=\frac{\delta Pr[y=j|x]}{\delta x_{l}}. \end{array}$$ (3)

The marginal effects represent the variation in the probability of picking one given response (*e.g*. “Agree”) if the individual is a woman. Marginal effects are computed for every possible response ranging from “Strongly Disagree” to “Strongly Agree”.

**Fig 3:**
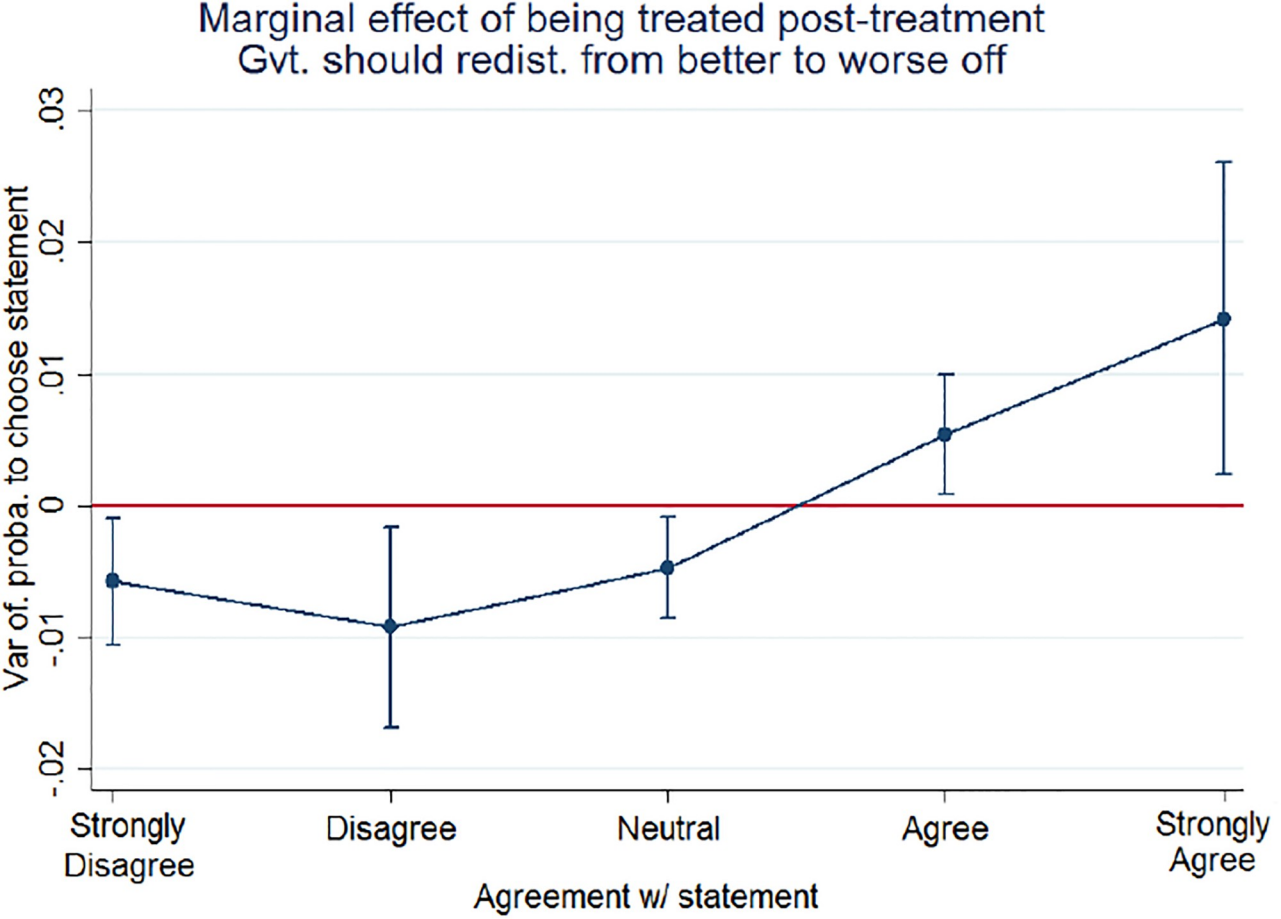
Marginal effects from ordered probit estimates (Source: BES data, own calculations).

Marginal increases in probabilities essentially indicate that post-leak, neutral and very positive outcomes are the most likely to occur. Fig 3, S5 and S6 Figs visually corroborate these conclusions for each statement on redistribution preferences. More precisely, I find an increase of 2.5% for the statement “Ordinary people do not get their fair share of the nation’s wealth” and an increase of 2% for the statement “Government should redistribute income from the better to the worse off”. Finally, after the scandal, agreement with the statement “Government should try to make income more equal” increases by 3.3%.

Many drivers of redistribution preferences have been explored in the literature and is at the centre of a review [13]. This subsection assesses the variation of responses to statements on redistribution after the Panama Papers scandal. The effects appear to be robust to income controls in regressions, thus indicating that individual reactions are not essentially driven by socio-demographics and economic variables.

### Heterogeneous effects

#### Heterogeneity on socio-demographics

This specification captures weak heterogeneous effects, as presented in Table 2, as well as S11 and S12 Tables. First, I observe no clear-cut gender heterogeneity. However, the observation of the interaction term yields a positive effect on redistribution preferences after the scandal for men. Second, coefficients do not record differentiated individual responses for any level of income.

**Table 2:** Heterogeneity of responses by socio-demographics and trust: “Government should try to make income more equal”.

VARIABLES	Dep. Var.: Government should try to make income more equal						
	Men	>50 y.o.	<£ 9,999 / year	>£ 10,000 & <39,999/year	>£ 40,000 / year	No trust in gvt.	Full trust in gvt.
	(1)	(2)	(3)	(4)	(5)	(6)	(7)
Post 3 Apr. 2016 (Yes = 1)	-0.0404	-0.0626*	-0.123**	-0.0677**	0.0244	-0.0728**	-0.142**
	(0.0331)	(0.0323)	(0.0528)	(0.0302)	(0.0439)	(0.0313)	(0.0621)
Informed (Yes = 1)	0.212***	-0.0411*	0.195***	0.0872***	0.185***	0.0774***	0.0852
	(0.0288)	(0.0217)	(0.0395)	(0.0214)	(0.0295)	(0.0219)	(0.0559)
**Post 3 Apr. 2016 & Informed (Yes = 1)**	**0.00652**	**0.0587** *	**0.0612**	**0.0364**	**-0.0383**	**0.0411**	**0.143** **
	**(0.0379)**	**(0.0326)**	**(0.0533)**	**(0.0339)**	**(0.0483)**	**(0.0352)**	**(0.0711)**
Time Controls	Yes	Yes	Yes	Yes	Yes	Yes	Yes
Controls socio dem	Yes	Yes	Yes	Yes	Yes	Yes	Yes
Control income	Yes	Yes	Yes	Yes	Yes	Yes	Yes
Observations	39,948	41,562	7,721	46,139	29,871	23,051	14,901

Standard errors are in parentheses and clustered at the interview date level.

*** p<0.01,

** p<0.05,

* p<0.1

Source: BES W13 Panel v1.2, own calculations

Question: How much do you agree or disagree with the statement: “Government should redistribute income from the better off to those who are less well off”?

Furthermore, individuals with high trust levels in Members of Parliament (MP) have higher preferences for redistribution: that is, they are more likely to trust the media and update their beliefs when receiving information on tax evasion. I find heterogeneous effects by trust levels and, more specifically, a positive and significant effect on those with higher trust levels (5 and above on a 10-point scale). These findings support the literature on preference updating and trust, namely literature which shows that low trust in the government leads to small effects for all other redistribution policies [6]. In other words, distrust in the government hinders people from transferring concern about inequality to support for redistribution by the government. The variation in responses is the most important for the perception of the legal system fairness.

#### Heterogeneity on political variables

Finally, I test for the existence of differentiated responses with respect to political affiliation. Table 3 as well as S13 and S14 Tables present the corresponding results. In the BES data, political affiliations are self-declared. Note that this defines general beliefs and not necessarily political parties. To verify the validity of the results, I ran complementary regressions on the stated newspapers read by the respondents. Individuals largely know about the existence and nature of newspapers’ political leaning (see [19]). From this information, it is possible to show that readers of *The Times* (respectively *The Guardian*) are more likely to define themselves as right-wing (respectively left-wing).

**Table 3:** Effects on redistribution preferences by political affiliation.

	Dep. Var.: Gvt. should redistribute from better to worse off			
	Pro Right-wing	Times	Pro Left-wing	Guardian
	(1)	(2)	(3)	(4)
Post Apr. 3rd, 2016 (Yes = 1)	-0.0790*	-0.210	-0.00290	0.127
	(0.0474)	(0.243)	(0.0772)	(0.167)
Informed (Yes = 1)	-0.152***	-0.236	0.309***	0.503***
	(0.0220)	(0.165)	(0.0556)	(0.115)
**Post Apr. 3rd, 2016 & Informed (Yes = 1)**	**0.108** ***	**0.264**	**-0.0925**	**-0.195**
	**(0.0380)**	**(0.252)**	**(0.0813)**	**(0.165)**
Time Controls	Yes	Yes	Yes	Yes
Controls socio dem	Yes	Yes	Yes	Yes
Control income	Yes	Yes	Yes	Yes
Observations	31,886	3,792	22,573	5,639

Standard errors are in parentheses and clustered at the interview date level.

*** p<0.01,

** p<0.05,

* p<0.1

Source: BES W13 Panel v1.2, ESS W8 v.1, own calculations.

Question: How much do you agree or disagree with the statement: “Government should redistribute income from the better off to those who are less well off”?

First, Column (3) in Table 3 shows no clear heterogeneous effects from left-wing individuals. Additional regressions on the Guardian readership in Column (4) confirm this result. Column (1) shows that those who declare themselves as “right-wing” experience a significantly greater update of their beliefs. This differential in responses by political affiliation is in line with studies that explain that the belief update is stronger among Republicans than Democrats as a result of a ceiling effect [9]. That is, most Democrats (and left-wing individuals in general) were supportive of welfare expansion even before the crisis. More precisely, for the statement “Government should redistribute income from the better to the worse off”, the average response of left-wing individuals is “agree” (*i.e*., 4 on a 5-point scale), while the average response of right-wing individuals is “neither agree nor disagree” (point 3). Similarly, the average response of Guardian readers is “agree” (point 4), while the average response of Times readers is “neither agree nor disagree” (point 3). This indicates that an informational shock can lead to a change in stated redistribution preferences and can also lead possibly to a change individuals’ social policy preferences, which corroborates previous findings in the literature [9].

These results highlight a difference in individual responses after the Panama Papers scandal. Right-wing voters increase their stated preferences for wealth redistribution, which indicates that it is necessary to check voting outcomes, namely stated voting intentions.

### Voting outcomes

As the previous section showed, individuals update their stated preferences after a scandal. However, does this effect translate into actions in general and in stated voting intentions more precisely?

Table 4 presents results on stated voting intentions for political parties. Additional information on tax evasion committed by top-income earners appears to bridge a gap between “right-wing” and “left-wing” individuals (see Columns (1) and (2)). In addition, stated voting intentions decrease for the UK Independence Party (UKIP). This result is in line with studies which find that information on inequality bridges the gap between individuals who have different preferences, materialised into different stated voting preferences [6]. The increase in stated voting intentions for the left and centre is in line with the call of Jeremy Corbyn (Labour leader) for ministers to publish their tax statements in the aftermath of the leak (see [20]), and Tim Farron’s (Liberal Democrats [LibDems] leader) statement that PM Cameron’s conduct is ‘morally murky’ (see [21].

**Table 4:** Effect of the Panama Papers scandal on stated voting intentions.

	Tories	Labour	LibDem	UKIP	Will Abstain	Dont Know
	(1)	(2)	(3)	(4)	(5)	(6)
Post 3 Apr. 2016 (Yes = 1)	0.130***	0.0536**	0.0803***	0.222***	-0.661***	-0.143***
	(0.0254)	(0.0228)	(0.0204)	(0.0215)	(0.0268)	(0.0233)
Informed (Yes = 1)	0.115***	-0.0551**	0.116***	-0.0629**	-0.0534	0.291***
	(0.0325)	(0.0274)	(0.0329)	(0.0284)	(0.0693)	(0.0661)
**Post 3 Apr. 2016 & Informed (Yes = 1)**	**-0.0748** **	**0.0474** *	**0.0434**	**-0.0528** *	**-0.0920** ***	**0.0424**
	**(0.0323)**	**(0.0287)**	**(0.0309)**	**(0.0271)**	**(0.0342)**	**(0.0324)**
Time Controls	Yes	Yes	Yes	Yes	Yes	Yes
Controls socio dem	Yes	Yes	Yes	Yes	Yes	Yes
Control income	Yes	Yes	Yes	Yes	Yes	Yes
Observations	81,292	81,233	80,846	82,108	120,010	120,010

Standard errors are in parentheses and clustered at the interview date level.

*** p<0.01,

** p<0.05,

* p<0.1

Source: BES W13 Panel v1.2, own calculations.

Estimates record a decrease in support for the Conservative party. This might be explained by the former prime minister’s (David Cameron) family being directly involved in the Panama Papers scandal. Although he did not run the party in 2017’s elections, his leadership might have affected respondents’ perception of the party and thus their answer during the survey.

The evolution of stated voting intentions over the course of the scandal shows a general move towards pro-redistribution parties and a decrease in abstention. This indicates that the scandal had an impact on potential switchers, though there is no variation from the undecided (Column (6) in Table 4). It appears that respondents tended to take a stand after the Panama Papers scandal. Results show a significant increase in stated voting intentions for Labour and a decrease for both the Conservatives, UKIP and people who decided they would abstain from voting. All these results put together indicate that the Panama Papers scandal encouraged a shift of stated voting intentions towards the left.

During the questionnaire, respondents were first presented with a list of parties and they state who they would vote for if there was a general election tomorrow. Then, they state on a scale ranging from 1 to 7 how certain they are they will vote for the party they previously mentioned (1-Not at all certain; 7-Completely certain). Estimates related to the certainty of stated voting intentions are presented in Table 5. Coefficients are only significant for the LibDem party. Thus, the change in stated voting intentions we observed for the Conservatives, Labour and UKIP parties are not necessarily strong and they are not accompanied by a change in the certainty of this decision. For LibDem voters, although individuals do not increase their propensity to vote for this party, the motivation of individuals to plan to vote for this party appears to be stronger post scandal. It is important to precise that the scandal affects only one dimension of the votes. Therefore, it is important to acknowledge that the scandal can affect voters, but without changing their actual voting behaviours as other parameters also influence one’s final voting decision.

**Table 5:** Effect of the Panama Papers on the certainty of the stated voting intention.

	Tories	Labour	LibDem	UKIP
	(1)	(2)	(3)	(4)
Post 3 Apr. 2016 (Yes = 1)	-0.220***	-0.0429	0.0499	0.288***
	(0.0371)	(0.0325)	(0.0327)	(0.0238)
Informed (Yes = 1)	-0.0270	0.0911*	-0.0388	-0.143***
	(0.0613)	(0.0549)	(0.0678)	(0.0390)
**Post 3 Apr. 2016 & Informed (Yes = 1)**	**0.0818**	**-0.0589**	**0.127** **	**0.0111**
	**(0.0603)**	**(0.0589)**	**(0.0613)**	**(0.0376)**
Time Controls	Yes	Yes	Yes	Yes
Controls socio dem	Yes	Yes	Yes	Yes
Control income	Yes	Yes	Yes	Yes
Observations	20,523	21,763	120,010	120,010

Standard errors are in parentheses and clustered at the interview date level.

*** p<0.01,

** p<0.05,

* p<0.1

Source: BES W13 Panel v1.2, own calculations.

### Robustness checks

The difference-in-differences strategy consistently captures a significant increase in redistribution preferences after the Panama Papers scandal. This strategy requires a common trend assumption between the treated and control groups. I test its validity next.

#### Placebo test

In order to test the validity and robustness of our results, I compute a placebo test. To do so, I use April 3rd, 2015 as the cutoff date. Estimates of the ordered probit placebo test are presented in S22 Table, and indicate that choosing another date yield no change in redistribution preferences, which is in line with the fact that Panama Papers are at the root of the change in responses. I also computed another placebo test assuming another false cutoff date, this time after the Panama Papers. S23 Table presents ordered probit estimates using December 3rd, 2016 as a cutoff. Results indicate that the interaction terms are not significant, which again corroborates that it is the Panama Papers scandal which initiated the change in stated preferences for redistribution.

#### Common trend test

First, I visually inspect pre-treatment trends in redistribution preferences to support the validity of the parallel trend assumption. S7 Fig graphically shows the common trend assumption. Second, I analyse the dynamic impact of the reform using a common trend test.

As a common trend test, I computed a test of lags and leads. This test contains all interaction coefficients capturing whether individuals are treated at each given period. Following the rule established in the literature, the aim of this test is to show that pre-treatment data presents a clear trend, which can then be applied to the post-scandal period (see [22]). In this study, the assumption that pre-treatment data establish a clear trend on average is represented by the F-test of joint insignificance of the leads. I expect the coefficients on leads to be jointly non-significant, which would indicate that being informed before the scandal has no impact on redistribution preferences. This works as a dynamic placebo test.

In this study, I chose the lags and leads precision level where one lag (respectively one lead) represents a given month, following common practice in the literature [23].

**Fig 4:**
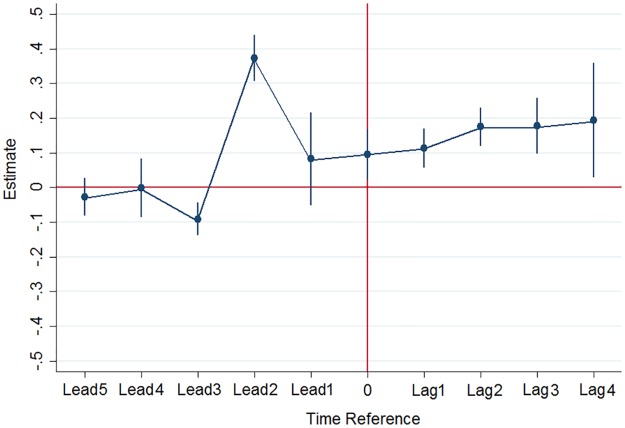
Common trend test estimates (lags and leads) (BES data).

I use the following specification:

$$y_{it}=\beta_{0}+\sum_{k}\beta_{i}^{k}x_{it}^{k}+\delta_{1}Post_{it}+\delta_{2}Informed_{it}$$ (4)

$$+\sum_{k}\gamma_{k}*Informed_{it}*Leads\_Lags_{k}+\epsilon_{it}$$ (5)

where *k* goes from −5 to 4 and defines the period used. First, the coefficients on the leads are jointly non-significant (∑_*k*_ *γ*_*k*_ = 0 if *k* < 0), and the coefficients on the lags are jointly significant (∑_*k*_ *γ*_*k*_ ≠ 0 if *k* > 0). These results indicate that this identification strategy truly identifies the impact of the Panama Papers scandal and does not pick up the effects of other elements that were affecting the treatment and control groups differently before the leak. Second, this complementary analysis rules out significant anticipation effects. The regressions allow for an implicit placebo test. Fig 4 shows the value of the coefficients *γ*_*k*_ for each period. The fact that lead 2 is significant and positive does not hinder in itself the validation of the common trend assumption since leads are jointly insignificant and that indicates that the common trend holds on average. Before April 3, 2016, being informed should not have had any impact on preferences for redistribution. In addition, when I examine the impact of the scandal for another statement, I find the same result (see S7 Fig).

We compute F-tests of the joint significance for the coefficients from the test of lags and leads. Results are presented in S21 Table. Estimates suggest that for redistribution statements, the leads are jointly insignificant and the lags are jointly significant. This is in line with the fact that the reaction of individuals is localised essentially after the Panama Papers scandal, which confirms that the common trend assumption holds.

#### Falsification tests

***General social and economic outcomes***. Falsification tests use the same specification as in Eq 1. Dependent variables are statements that cover the political action on matters different from redistribution preferences: (i) “Do you agree with the government policies towards immigration”? (ii) “Did measures to protect the environment go too far”? and (iii) “What do you think of the death penalty”? Individuals choose their answer on a 5-point scale (1 = completely disagree, 5 = completely agree).

S19 Table presents the falsification tests’ results for the main outcomes. The results show that the opinion/preferences of individuals on side topics such as immigration, the environment and the death penalty do not significantly change after the Panama Papers scandal. This result highlights the redistribution channel as the main channel for the update of beliefs after a scandal.

**EU and Brexit-related outcomes**. The Brexit debate brought about intense political debates at a time that was relatively close to that of the Panama Papers. Hence, it is necessary to include falsification tests for outcomes that are related to the Brexit debate. These tests are crucial to prove that the change in individual responses that are recorded are not a side effect of the Brexit debates. Research into the main hot topics related to the Brexit campaign indicate the debate showed issues like: (i) The EU threatens British sovereignty; (ii) The EU is strangling the UK in burdensome regulations (iii) The EU entrenches corporate interests and prevents radical reforms (iv) The EU was a good idea, but the euro is a disaster (v) The EU allows too many immigrants (vi) The UK could have a more rational immigration system outside the EU, (vii) The UK could keep the money it currently sends to the EU (see [24] for the detailed list of arguments).

Outcomes selected for falsification tests are individual responses to a statement related to the perception of the European Union. Each one of them can be linked to a Brexit campaign argument. The selected arguments are the following: “Being a member of the European Union undermines Britain’s distinctive identity” (to test for argument (i) above), “European courts should be able to make decisions about human right cases” (to test for argument (i) above), “Some laws are better made at the European level” (to test for argument (ii)), “Good or bad for Britain: A Common European Agricultural Policy” (argument (iii) and (vii)), “Good or bad for Britain: joining a single European currency” (argument (iv)), “Good or bad for Britain: Allowing the free movement of workers within Europe” (argument (v), (vi)).

S20 Table presents estimates for EU-related falsification tests. All estimates are insignificant. This corroborates the fact that the changes in redistribution preferences are caused by the Panama Papers, and are not a response to the Brexit campaign.

#### Definition of the control and treated groups

The identification strategy comprises a definition of being informed. Here, being informed means perusing all media channels and being updated by other individuals. However, it is crucial to test whether the effects captured thus far are stable to the definition of being informed. This motivates the implementation of a robustness check, in which I modify the definition of the treatment (“being informed”): individuals in the treated group follow current affairs and politics via at least one of the media channels (television, radio, newspapers, or Internet). Table 6 presents the corresponding results.

**Table 6:** Ordered probit estimates: Alternative treated group (different definition of being informed).

	Government should…		Ordinary people do not get their fair share of nation’s wealth
	…redist. from better to worse off	…try to make income more equal	
	(1)	(2)	(3)
Post 3 Apr. 2016 (Yes = 1)	-0.120***	-0.0906***	-0.135***
	(0.0238)	(0.0317)	(0.0238)
Informed (Yes = 1)	0.0242	-0.0709***	0.0733***
	(0.0148)	(0.0172)	(0.0181)
**Post 3 Apr. 2016 & Informed (Yes = 1)**	**0.0410** *	**0.0744** ***	**0.0808** ***
	**(0.0236)**	**(0.0263)**	**(0.0257)**
Time Controls	Yes	Yes	Yes
Controls socio dem	Yes	Yes	Yes
Control income	Yes	Yes	Yes
Observations	63,293	94,035	63,921

Standard errors are in parentheses and clustered at the interview date level.

*** p<0.01,

** p<0.05,

* p<0.1

Source: BES W13 Panel v1.2, own calculations.

The results show a difference-in-differences effect that is maintained under this new definition of being informed. The effect is larger than that in Table 1.

#### Testing responses to treatment intensity

Additional specifications have been computed that accounts for different degrees of information respectively results for individuals who are informed on politics and current affairs (i) for at least 30min, (ii) for at least 1 hour and (iii) for at least 2 hours. Estimates are presented in S15 Table, S16 and S17 Tables. Results indicate that the longer individuals spend getting information, the more likely they are to change their preferences for redistribution. In addition, when respondents are less exposed to information, we are more likely to see no significant response of individuals. This is in line with the fact that information takes more time to be processed and to lead to a change in stated preferences.

Finally, we test whether responses differ in magnitude when individuals are informed through internet or through traditional media. S18 Table presents triple-differences regression estimates that include an interaction term between the treated group and individuals only informed via the internet.

## European comparisons

### Data

In this section, I use a cross-sectional dataset from the ESS. The survey includes 22 European countries; it amounts to 100,322 total observations over the 2014–2016 period. It contains information on wealth redistribution preferences through statements on which individuals give their opinion on a 5-point scale (1 = strongly disagree, 5 = strongly agree). It also has variables on the level of information. Respondents are asked how much time they spend per week reviewing news about politics and current affairs and whether they tend to watch, read or listen to news.

### Consistency at the european level

First, I check whether the Panama Papers scandal had (similar) effects on redistribution preferences at the European level. In this subsection, estimated regressions follow Eq 1 for a redistribution statement available for both the ESS and BES: “Government should try to make income more equal”. S25 Table presents the corresponding results. Column (1) shows the effects are comparable to the UK panel results in Table 1. Note that the magnitude of the effect is larger in the ESS than in the BES dataset; this is because both datasets have different structures. Columns (2) and (3) show that after the leak, the treatment generates a positive effect on stated redistribution outcomes across all European countries. This is in line with the literature showing that redistributive preferences may have “cultural” determinants that are stable over time [25]. The results are stable to the inclusion of country dummies (see Columns (2) and (3)). This constitutes proof that the reaction is consistent among individuals and robust to country heterogeneity. There may be a difference in the base level of redistribution preferences by country, but this variation after the leak is not affected much by country-specific differences in preferences.

Second, I conduct a falsification test on trust outcomes. The trust variable shares the same label both in the BES and ESS datasets; S24 Table suggests no effect of the Panama Papers scandal on trust levels.

### Cross-country comparisons

At the time of the leak, European media noted 124 personalities from various backgrounds (e.g., politics, music, sports) involved in the scandal. The distribution of these individuals by category is available in S2 Table. Yet, for some EU countries, no local personalities were mentioned. These countries included Albania, Bulgaria, Croatia, Cyprus, Czech Rep, Lithuania, Norway, Portugal, Slovakia, Slovenia, and Turkey; as such, it is useful to test for heterogeneous effects depending on whether the country is involved in the leak. I test for a differentiated effect between countries where at least one citizen was cited in the media as having been part of the Panama Papers scandal.

### Magnitude of the scandal by country and media coverage

In this subsection, estimated regressions slightly change from the previous specification. I use a difference-in-differences specification in which I test for the existence of differentiated responses in countries where at least one person is involved in the scandal. In this specification, the term of interest is the interaction term recording the response of treated (informed) post-scandal with respect to the media coverage and the extent to (i) how much was the country involved in the scandal and (ii) the media coverage intensity in the country.

Countries’ involvement in the Panama Papers is measured by the total number of individuals involved in the Panama Papers’ leak in Europe, from all sources: government, business, culture and art, sport, and other. Statistics on the country’s involvement is presented in S2 Table. The media coverage intensity is computed using the number of headlines in a country’s newspaper using the Factiva database. Media coverage intensity descriptive statistics are presented in S3 Table.

Column (1) of S26 Table shows no heterogeneity in responses between countries with at least one individual in the scandal and those with no individuals. However, Column (2) captures a larger increase in stated redistribution preferences for countries that have more citizens involved in the scandal. This effect results from two potential means: (i) perceptions of the individual involved in the leak or (ii) the intensity of the media coverage. Column (3) indicates the impact of media coverage and suggests that the greater the media coverage, the larger is the increase in stated preferences for redistribution after the leak. Note that the press coverage is greater in countries where personalities were named and involved in the leak (see S4 Fig). The press coverage in this specification is represented by the number of press article in the media.

In addition, Column (4) in S26 Table indicates that the pro-redistribution adjustment is higher in countries in which individuals are involved in the scandal and where the media coverage of the leak is more rigorous. The more individuals are involved in the scandal in a given country, the more citizens are in favour of redistribution in the country. In addition, conjugated increased media coverage magnifies this change in stated preferences.

## Conclusion

This study tests whether individuals revised their beliefs after the Panama Papers scandal, which provided information on tax evasion from top-income earners. I find that this informational shock influences individual beliefs. Stated redistribution preferences increase, and individuals also perceive the legal system as less fair. Estimates using ordered probit, OLS and panel fixed effects models yield consistent results.

Estimates suggest that individual reactions differ in terms of both age and political affiliation. I still note mild heterogeneity based on income levels. I test for heterogeneity by political affiliation: estimates indicate that right-wing individuals update their beliefs more than left-wing individuals. As mentioned, this is because left-wing individuals are more subject to a ceiling effect than the right-wing; the latter are more likely to move their preferences for redistribution upward.

In addition, I assess the effects of the scandal on voting outcomes. I test for potential dissonance in stated voting intentions (*i.e*., whether partisans remain loyal to their party). I find that the scandal incentivises more right-wing individuals into revising their beliefs. Fewer individuals mention they want to abstain from voting. Voting intentions increase for the left and decrease for the right. These results suggest that, in general, the scandal also affects perceptions of political parties. Fiscal scandals seem to encourage individuals with no clear voting intentions to take a stand in favour of more pro-redistribution parties. Yet preferences for redistribution only constitute one dimension of voting behaviour, which would explain why the impact of the Panama Papers scandal on voting intentions remains moderate.

Complementary estimations at the European level show consistent results, even when clearing the effects from country heterogeneity. Cross-country comparative analyses show that shock intensity (i.e., the number of individuals involved) and media coverage intensity increasingly affect the propensity to react to the scandal.

This analysis corroborates results from recent literature on the elasticity of preferences for wealth redistribution [6]. I also find that an informational shock triggers a change in individuals’ beliefs towards redistribution. As such, this study contributes to the literature by serving as a complement to the study of firm responses to informational leaks.

Tax havens generate inequality, which leads to the question of the optimality of taxation systems. However, the design of optimal taxation policies does not include the possibility that some individuals resort to offshore firms. Yet this study shows that individuals are sensitive to the existence of inequality and update their stated preferences accordingly. Therefore, the observation of the reaction of individuals to information related to taxation systems advocates for the inclusion of these behaviours in the design of optimal policies.

## Supporting information

S1 Fig Publications in UK newspapers containing “Panama Papers” keyword in 2016, by newspaper (Source: Factiva). (PNG)

S2 Fig Number of UK newspaper publications containing “Panama Papers” keyword over 2016 (Source: Factiva). (PNG)

S3 Fig Timeline of the main political events in the United Kingdom, 2009–2016. (PNG)

S4 Fig Average number of publications containing “Panama Papers” keyword in European newspapers in 2016, by country involvement (Source: Factiva). (PNG)

S5 Fig Marginal effects from ordered probit estimates: “Government should try to make income more equal” (Source: BES data, own calculations). (PNG)

S6 Fig Marginal effects from ordered probit estimates: “Ordinary working people do not get fair share of nation’s wealth” (Source: BES data, own calculations). (PNG)

S7 Fig Common trend test estimates (lags and leads): “Government should redistribute from the better to the worse off” (Source: BES data, own calculations). (PNG)

S1 Table ICIJ reporting partners in Europe (source: ICIJ). (PNG)

S2 Table Number of individuals involved in the Panama Papers scandal leak in Europe, by country (Source: ICIJ). (PNG)

S3 Table Media coverage descriptive statistics—Number of “Panama Papers” headlines in 2016, by country. (PNG)

S4 Table Press coverage of the Panama Papers scandal in the sun over year 2016 (selection). (PNG)

S5 Table Summary statistics and comparison with census data. (PNG)

S6 Table Summary statistics of the sample, split by informed and uninformed individuals. (PNG)

S7 Table Sample summary statistics: Statement responses and share of treated. (PNG)

S8 Table Ordered probit estimates: Effect of socio-economic variables. (PNG)

S9 Table Effect of the Panama Papers scandal on preferences: OLS estimates. (PNG)

S10 Table Effect of the Panama Papers scandal on preferences: Panel estimates. (PNG)

S11 Table Heterogeneity of responses by socio-demographics and trust: “Government should try to make income more equal”. (PNG)

S12 Table Heterogeneity of responses by socio-demographics and trust: “Ordinary working people do not get their fair share of the nation’s wealth”. (PNG)

S13 Table Effects on redistribution preferences by political affiliation: “Gvt. should try to make income more equal”. (PNG)

S14 Table Effects on redistribution preferences by political affiliation: “Government should redist. from the better to the worse off”. (PNG)

S15 Table Ordered probit estimates: Alternative treatment definitions—Exposure to information for 30mns and above. (PNG)

S16 Table Ordered probit estimates: Alternative treatment definitions—Exposure to information for 1 hour and above. (PNG)

S17 Table Ordered probit estimates: Alternative treatment definitions—Exposure to information for 2 hours and above. (PNG)

S18 Table Ordered probit estimates: Heterogeneity of responses with respect to technology (internet). (PNG)

S19 Table Falsification tests on preferences for redistribution: Main outcomes. (PNG)

S20 Table Falsification tests: Stated preferences for EU-related questions. (PNG)

S21 Table Test of joint significance for the statement—“Ordinary people do not get their fair share of people’s wealth” (BES data). (PNG)

S22 Table Ordered probit estimates: Alternative treated group—Placebo date—Before Panama Papers. (PNG)

S23 Table Ordered probit estimates: Alternative treated group—Placebo date—After Panama Papers. (PNG)

S24 Table Trust in government (OLS): Dependent variable. (PNG)

S25 Table Effect of the Panama Papers scandal on redistribution preferences: Validated at the EU level (OLS). (PNG)

S26 Table Effect of scandal intensity and media coverage by country on redistribution preferences. (PNG)
