# Supplementary figures and images for: Effects of signalling tax evasion on redistribution and voting preferences: Evidence from the Panama Papers
Source: PLoS One. 2020 Mar 10;15(3):e0229394. doi: 10.1371/journal.pone.0229394 (PMC7064205; doi:10.1371/journal.pone.0229394)

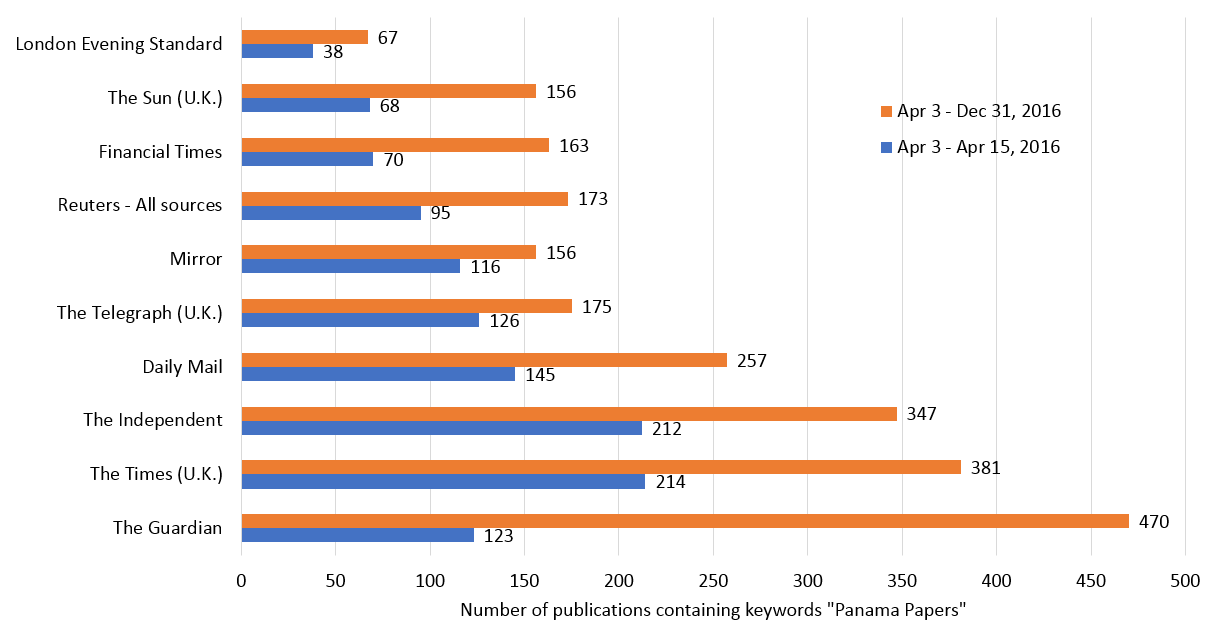

Supplement: S1 Fig — (PNG) [file pone.0229394.s001.png]

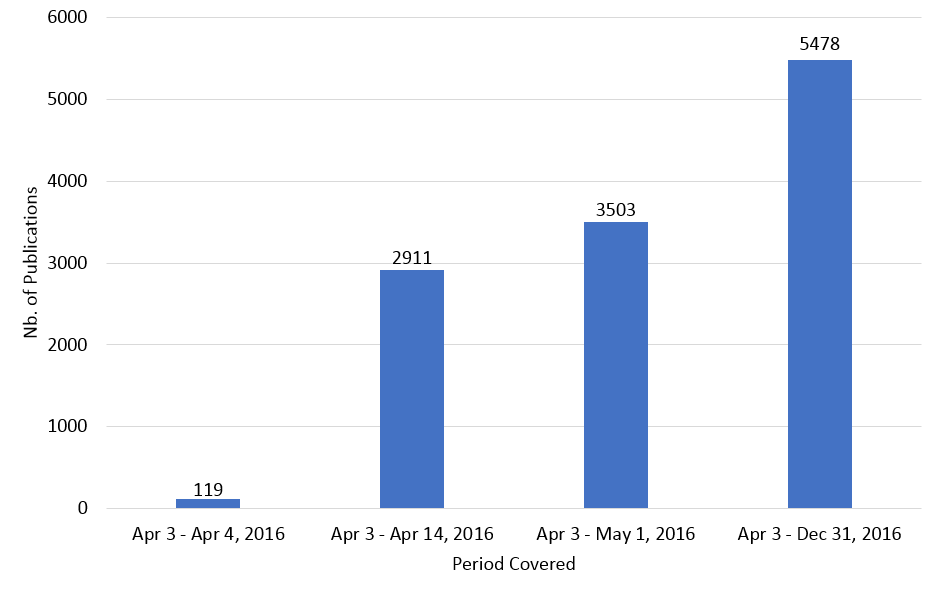

Supplement: S2 Fig — (PNG) [file pone.0229394.s002.png]

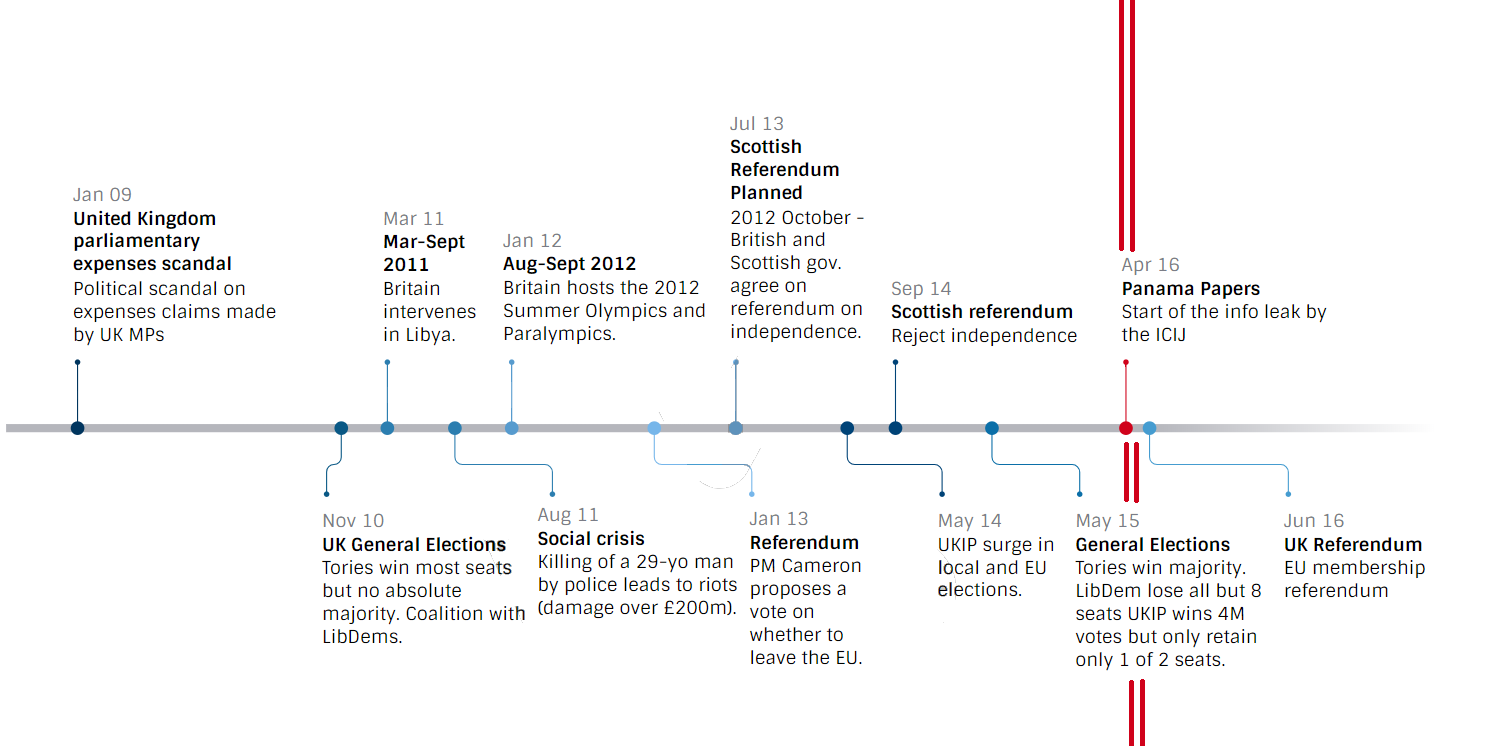

Supplement: S3 Fig — (PNG) [file pone.0229394.s003.png]

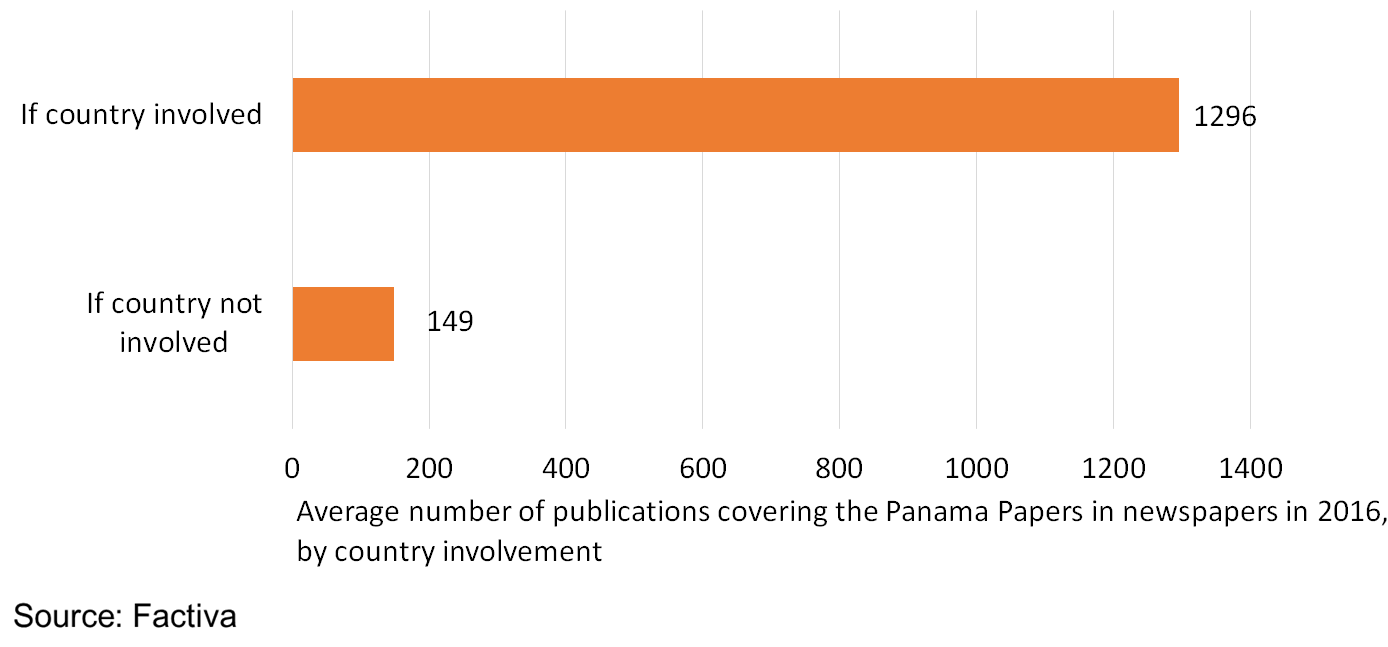

Supplement: S4 Fig — (PNG) [file pone.0229394.s004.png]

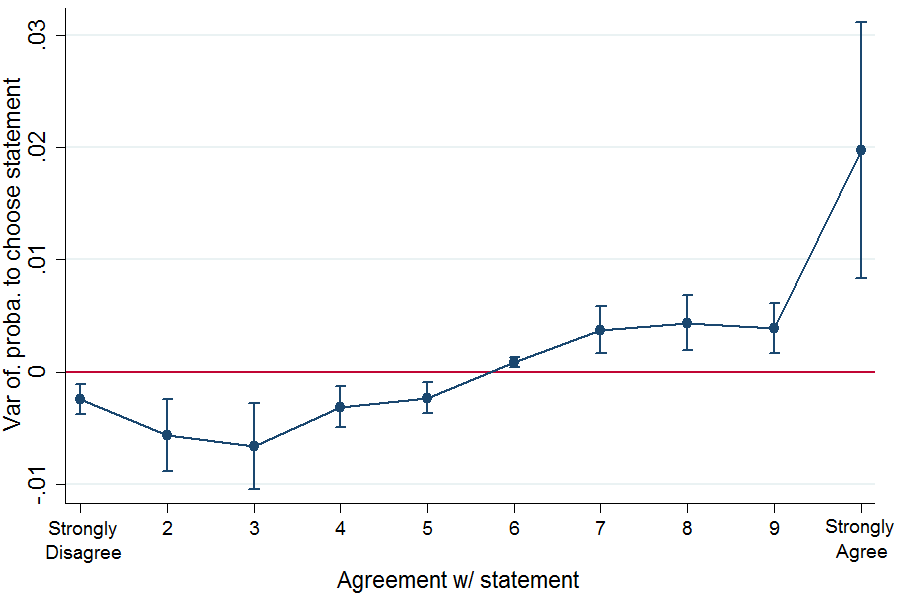

Supplement: S5 Fig — (PNG) [file pone.0229394.s005.png]

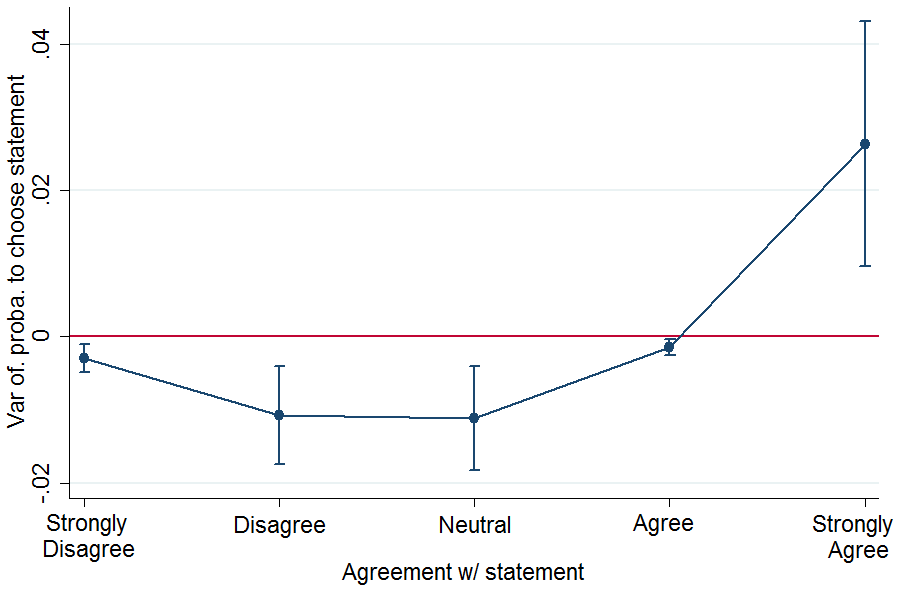

Supplement: S6 Fig — (PNG) [file pone.0229394.s006.png]

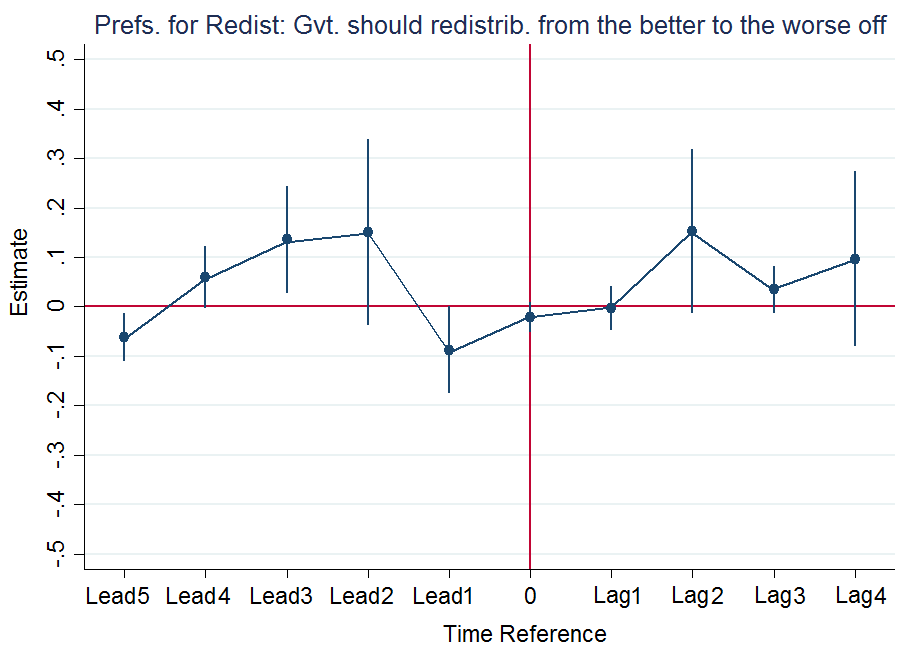

Supplement: S7 Fig — (PNG) [file pone.0229394.s007.png]

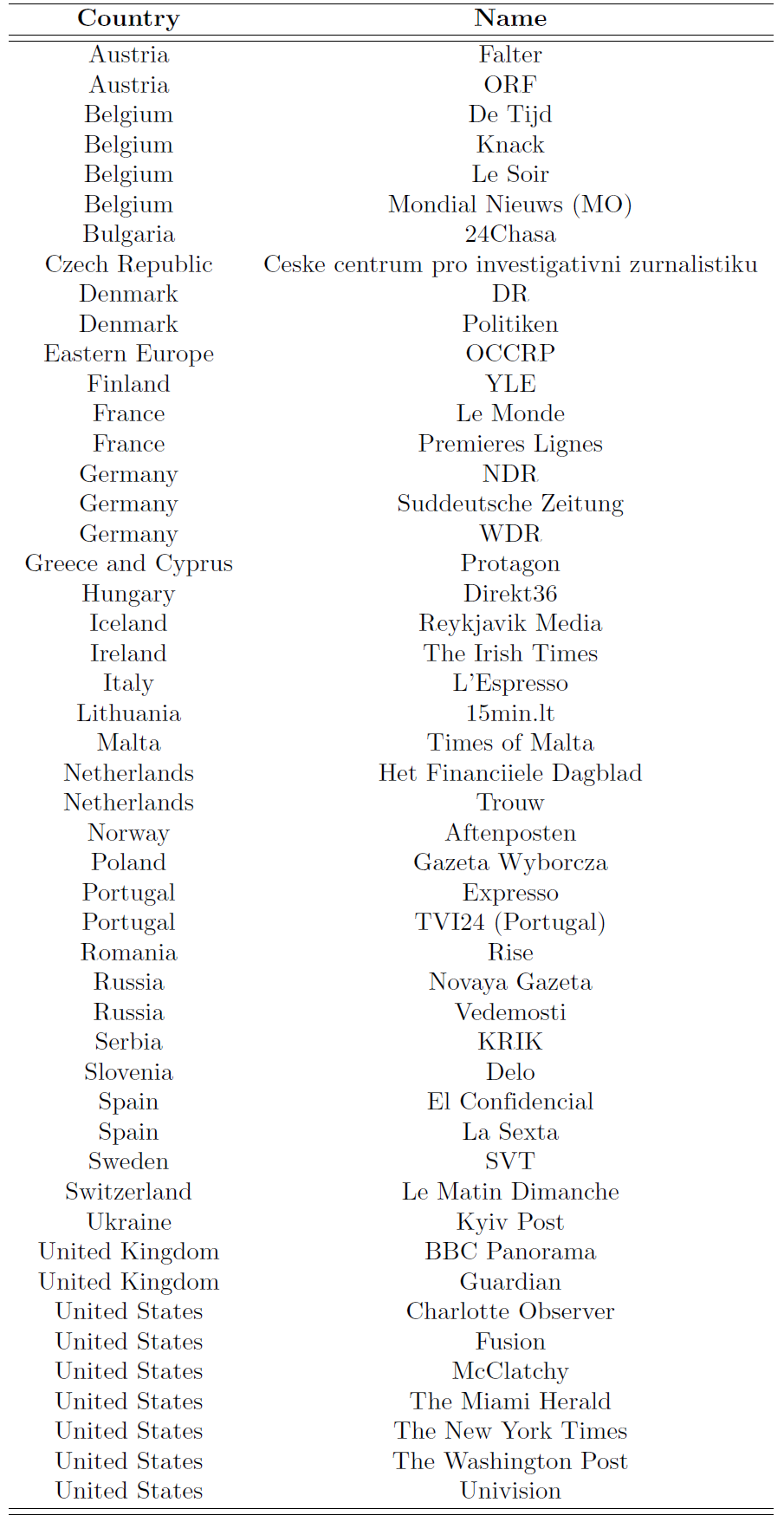

Supplement: S1 Table — (PNG) [file pone.0229394.s008.PNG]

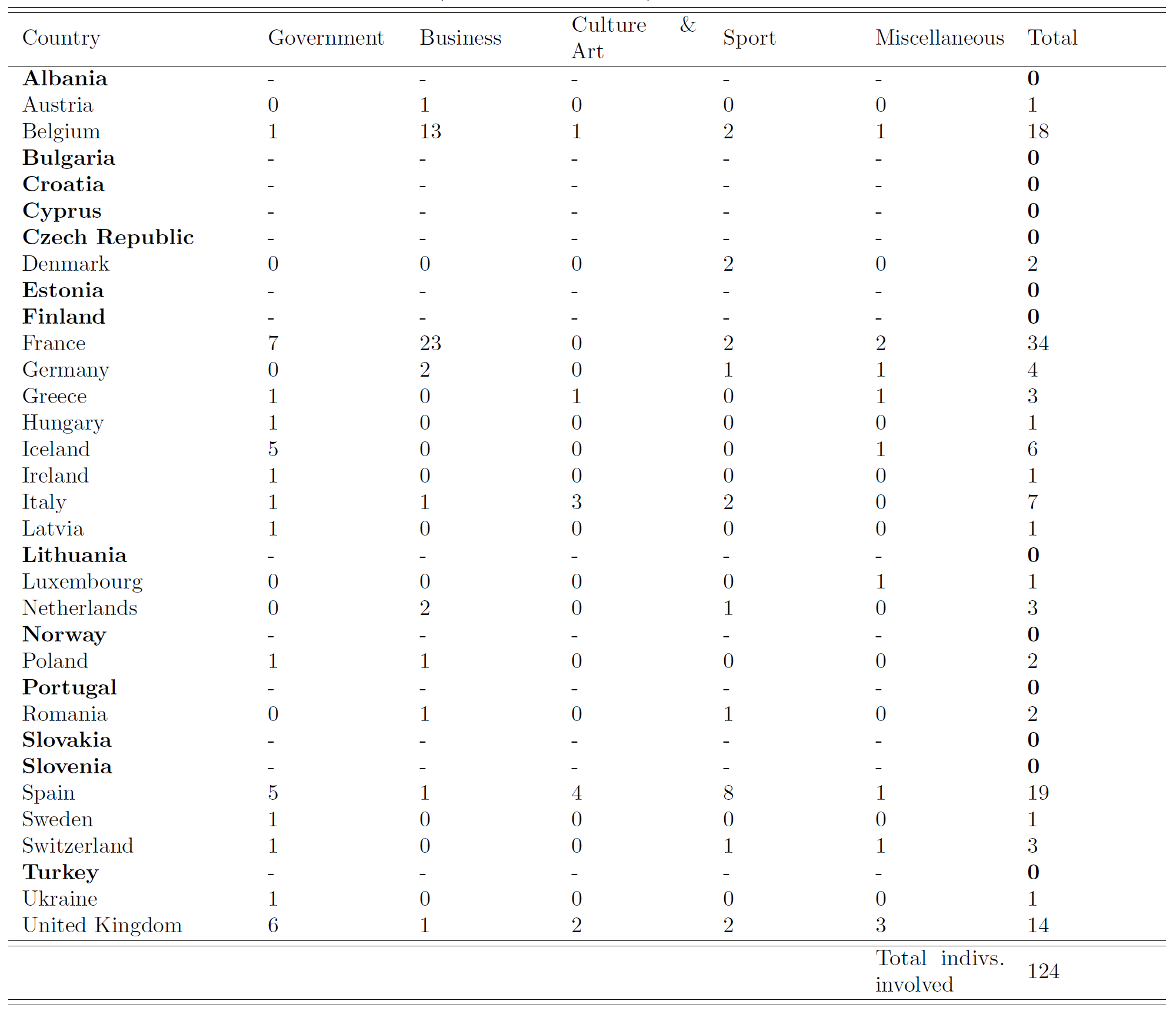

Supplement: S2 Table — (PNG) [file pone.0229394.s009.PNG]

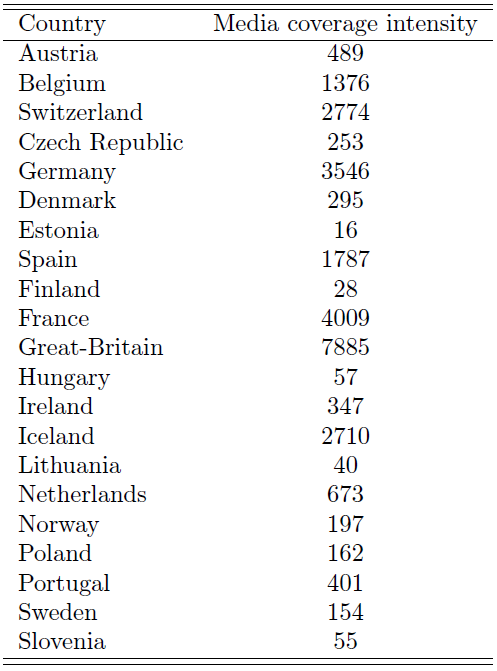

Supplement: S3 Table — (PNG) [file pone.0229394.s010.PNG]

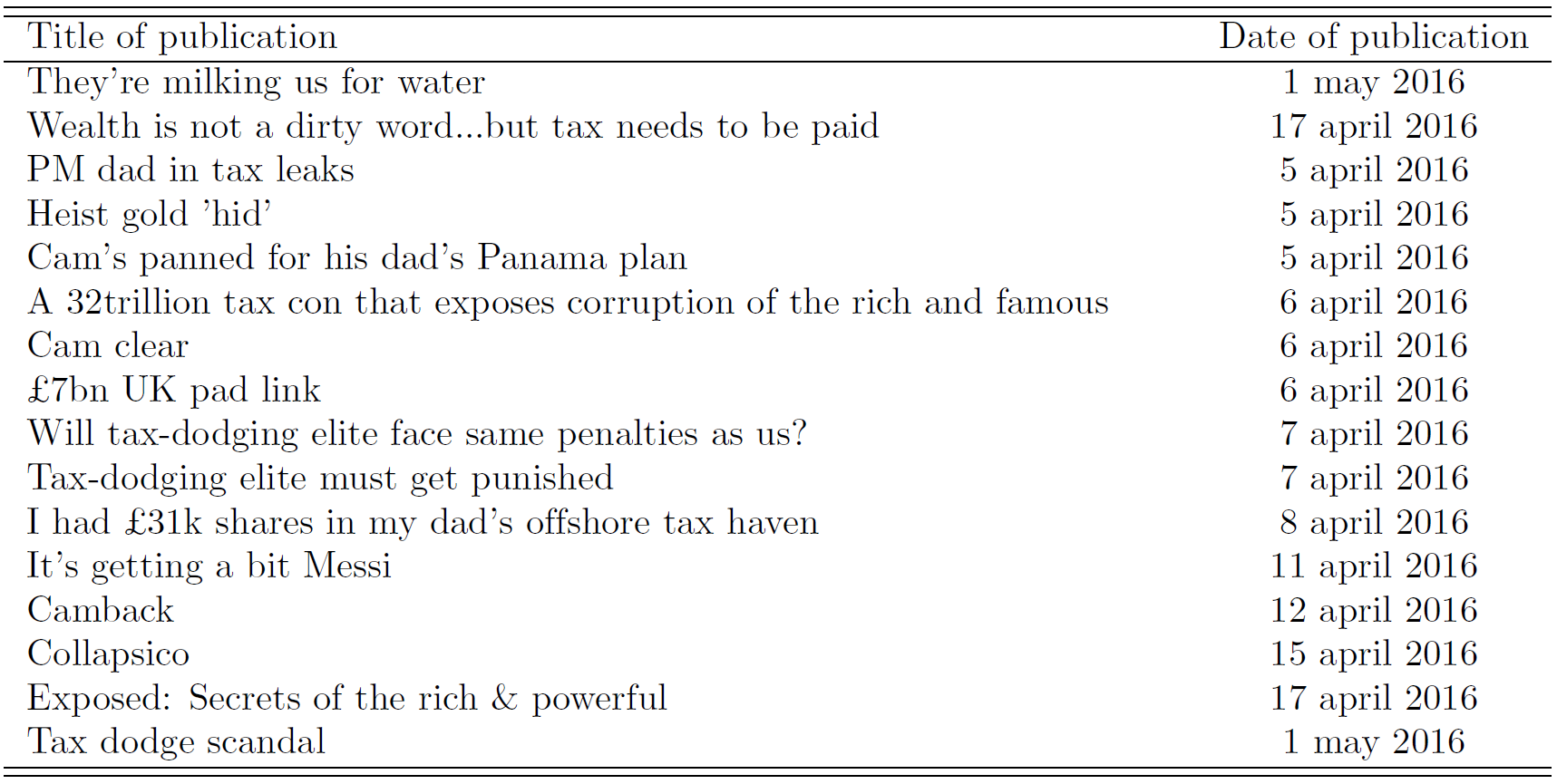

Supplement: S4 Table — (PNG) [file pone.0229394.s011.PNG]

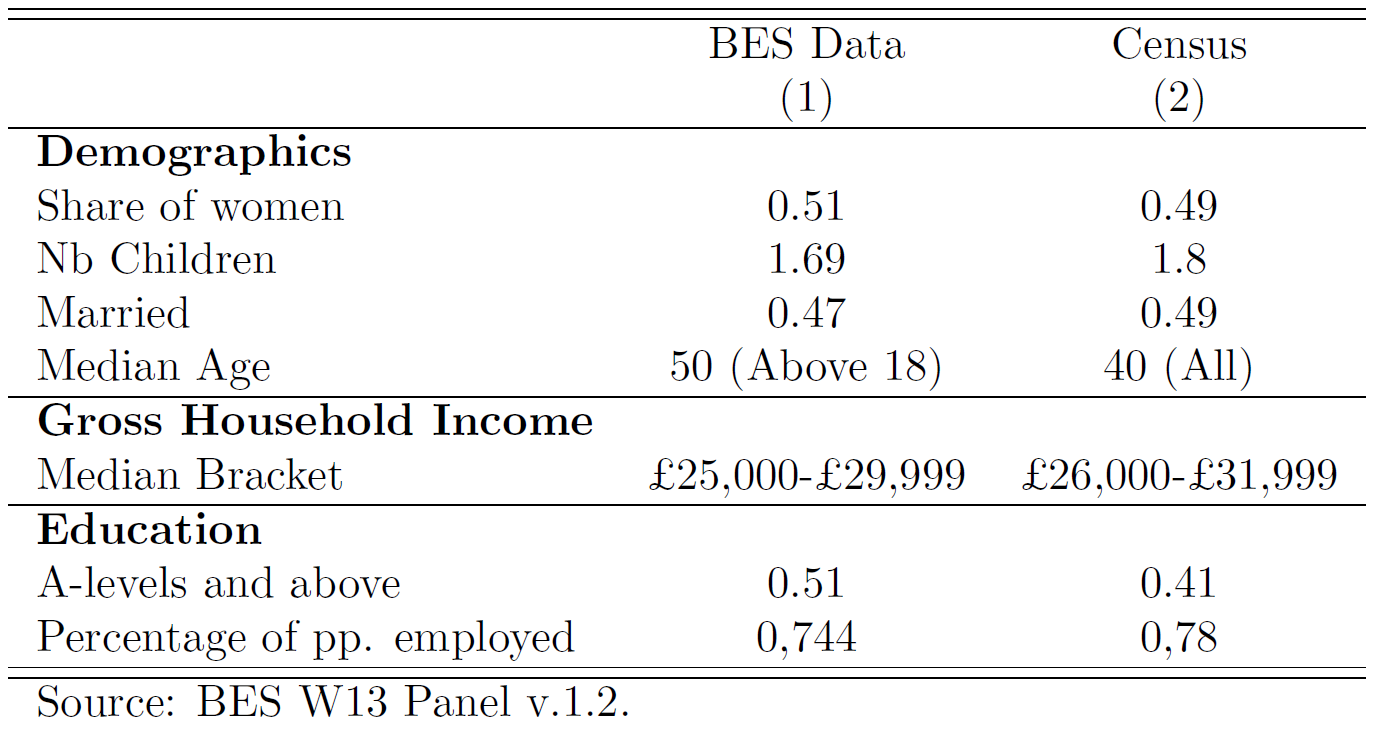

Supplement: S5 Table — (PNG) [file pone.0229394.s012.PNG]

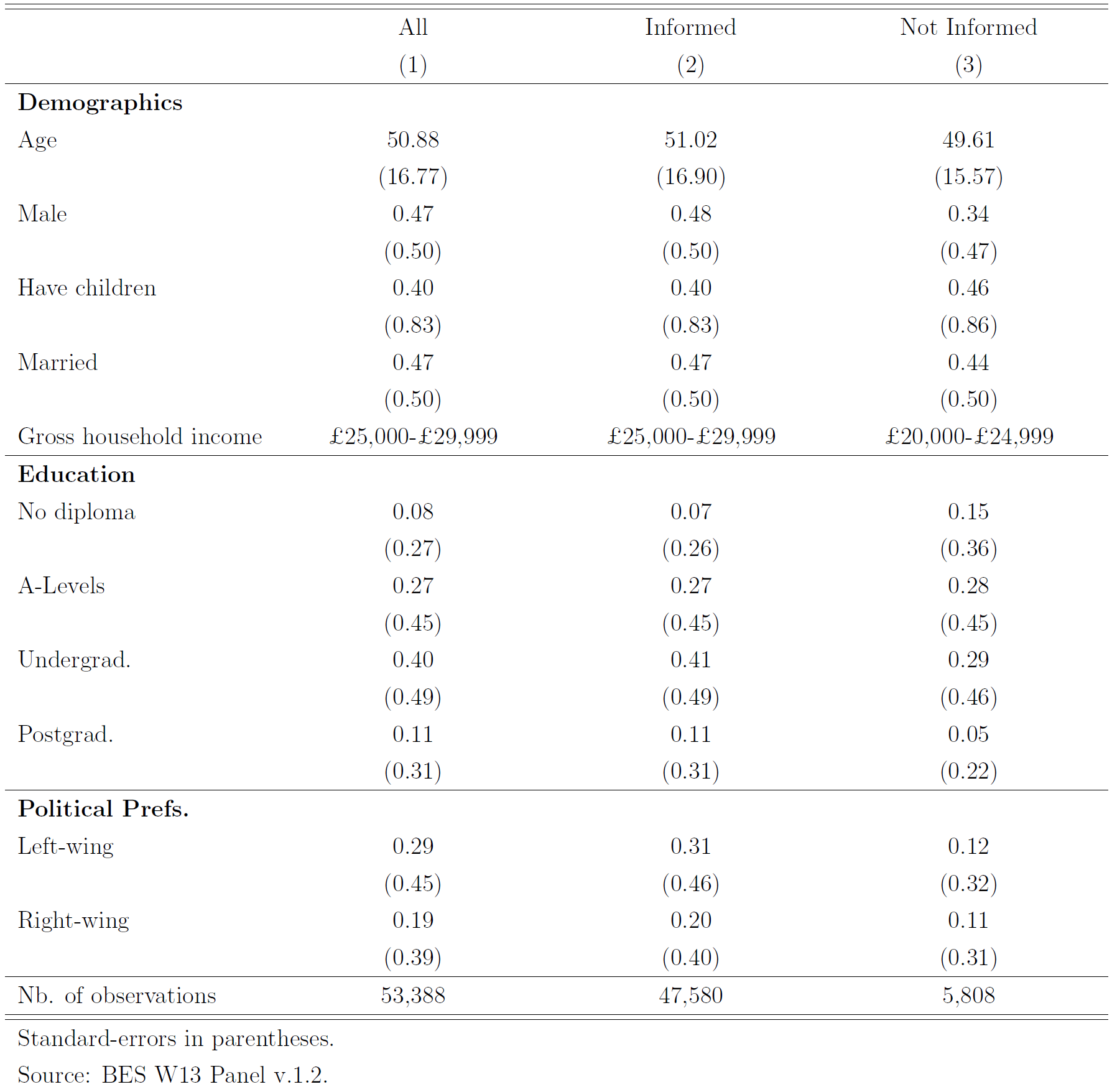

Supplement: S6 Table — (PNG) [file pone.0229394.s013.PNG]

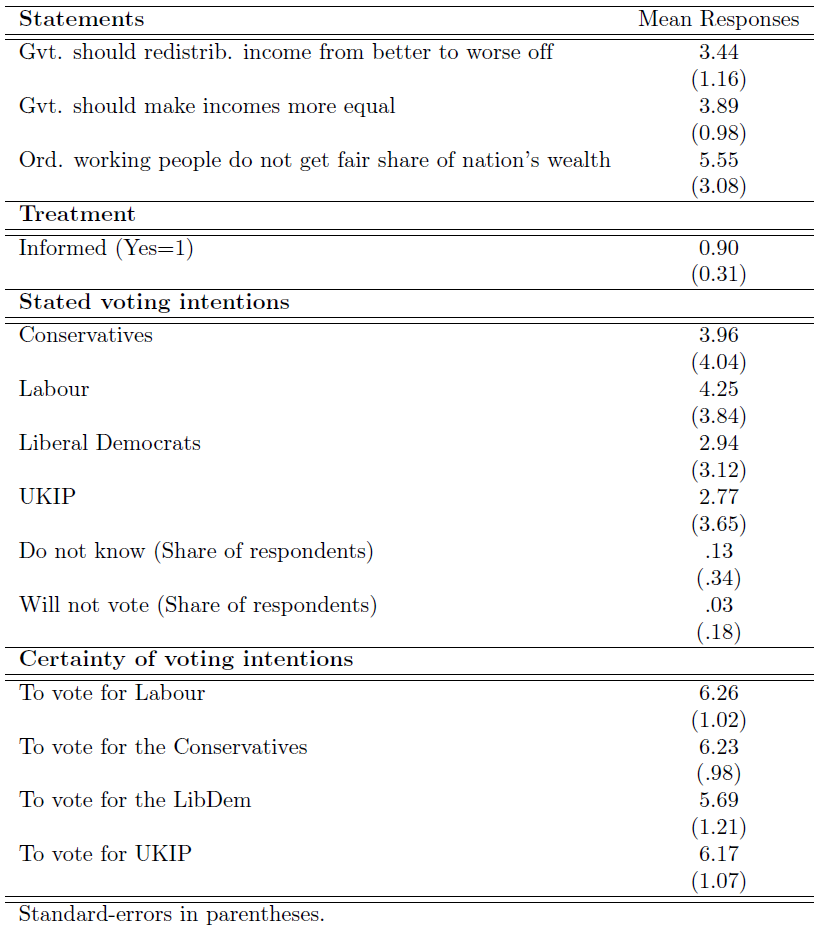

Supplement: S7 Table — (PNG) [file pone.0229394.s014.PNG]

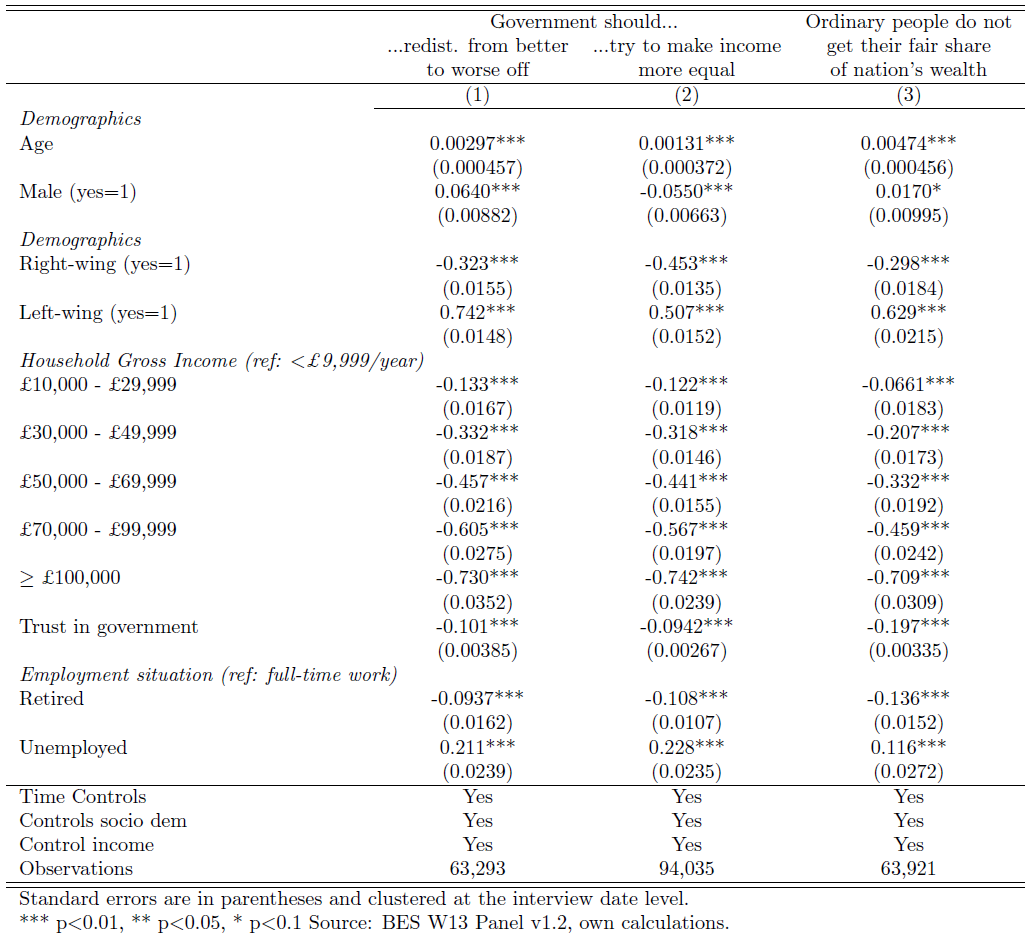

Supplement: S8 Table — (PNG) [file pone.0229394.s015.PNG]

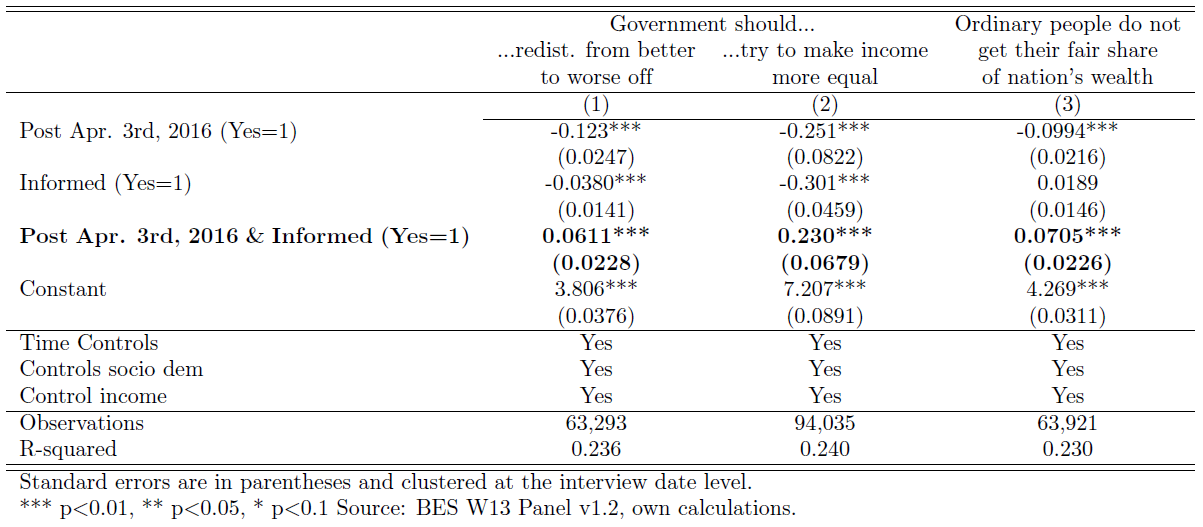

Supplement: S9 Table — (PNG) [file pone.0229394.s016.PNG]

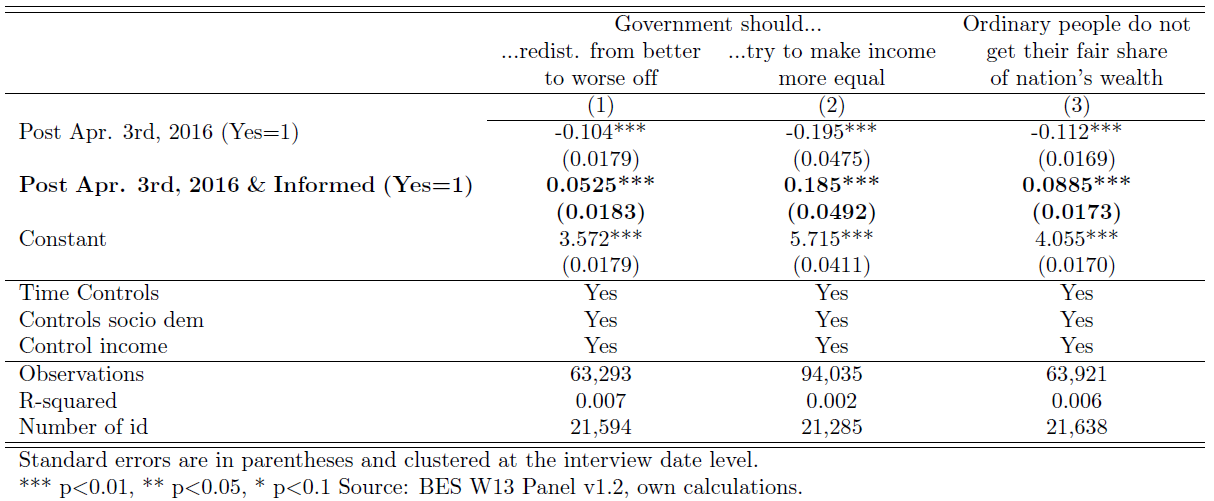

Supplement: S10 Table — (PNG) [file pone.0229394.s017.PNG]

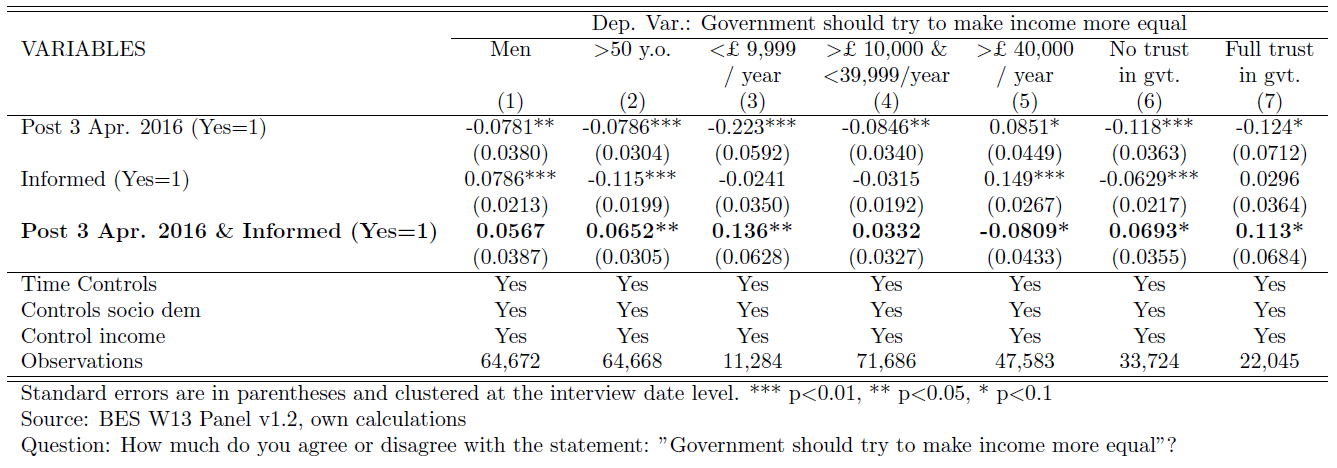

Supplement: S11 Table — (PNG) [file pone.0229394.s018.PNG]

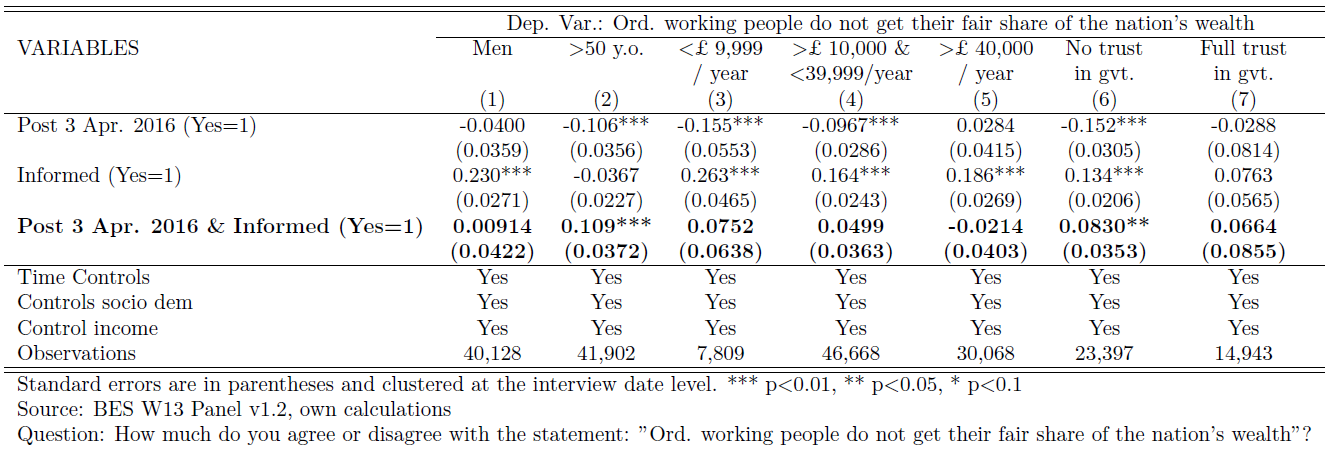

Supplement: S12 Table — (PNG) [file pone.0229394.s019.PNG]

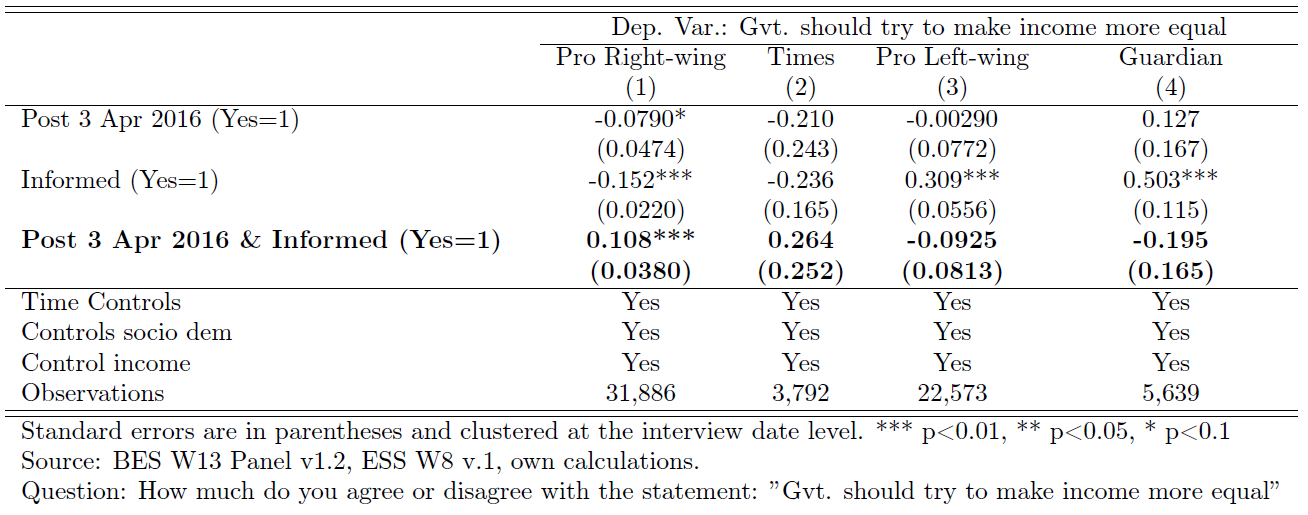

Supplement: S13 Table — (PNG) [file pone.0229394.s020.PNG]

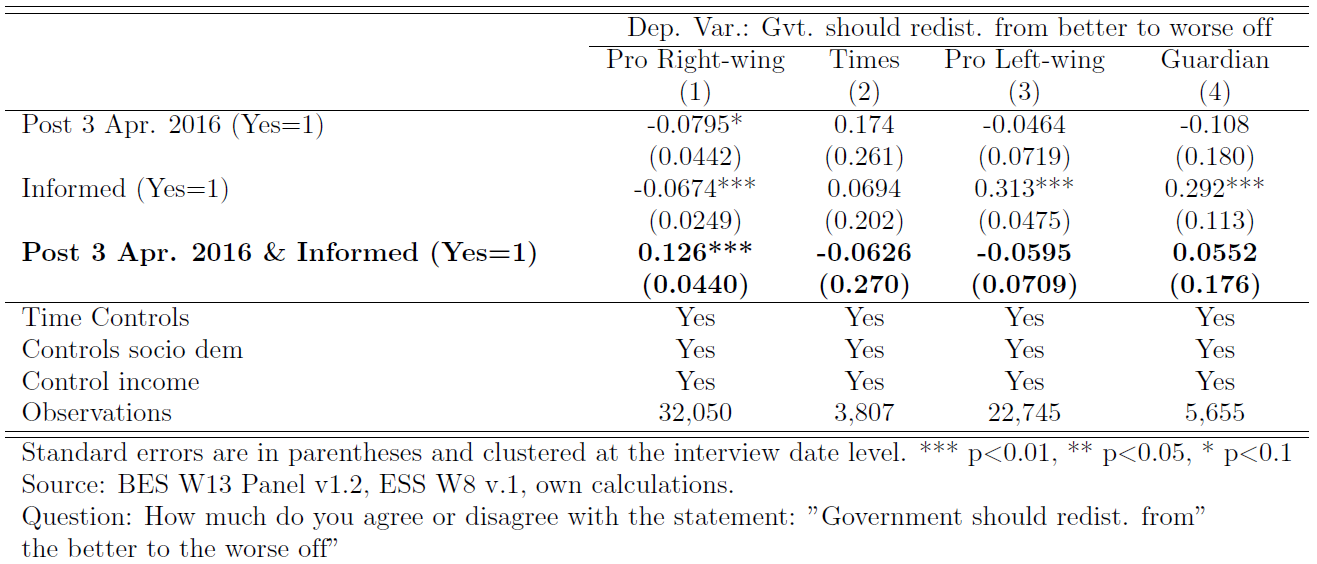

Supplement: S14 Table — (PNG) [file pone.0229394.s021.PNG]

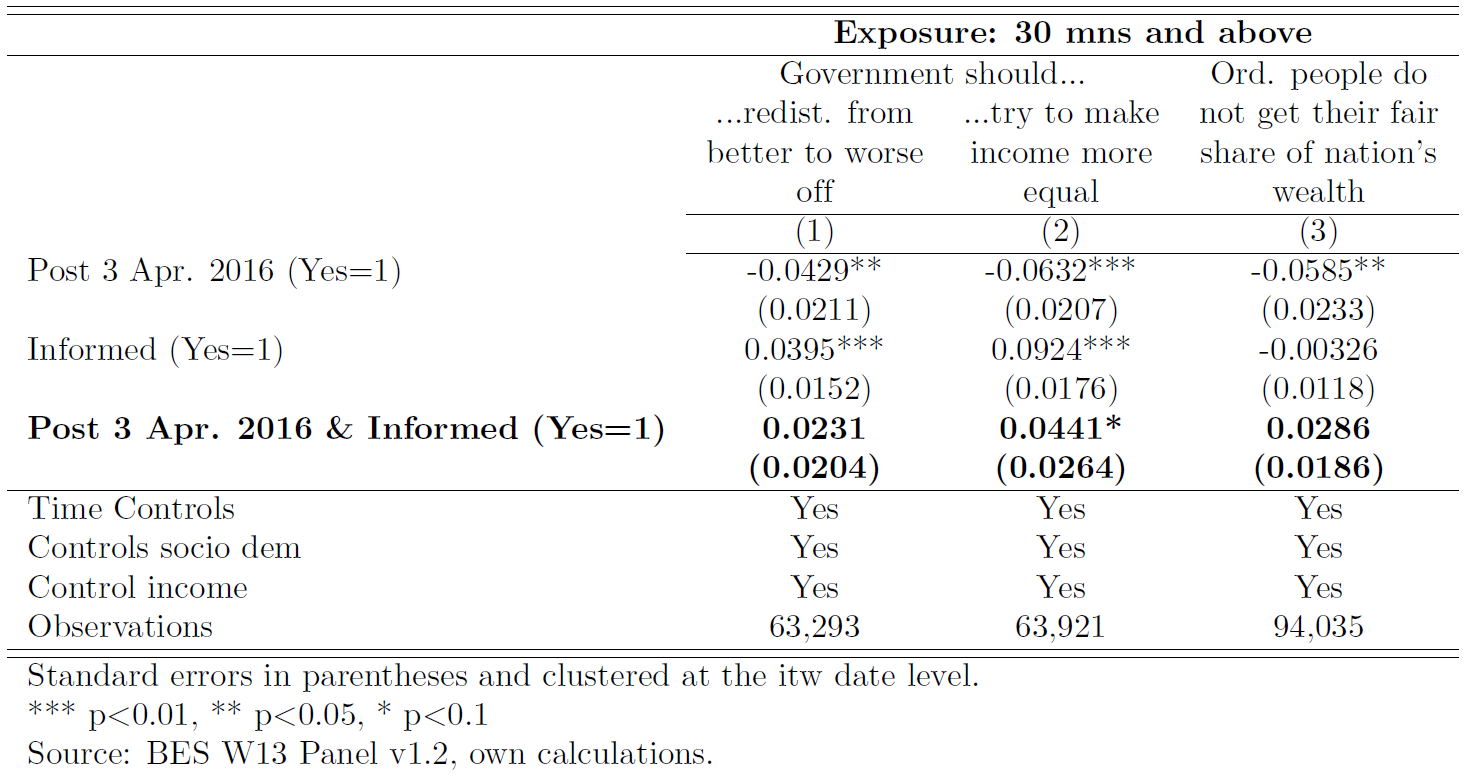

Supplement: S15 Table — (PNG) [file pone.0229394.s022.PNG]

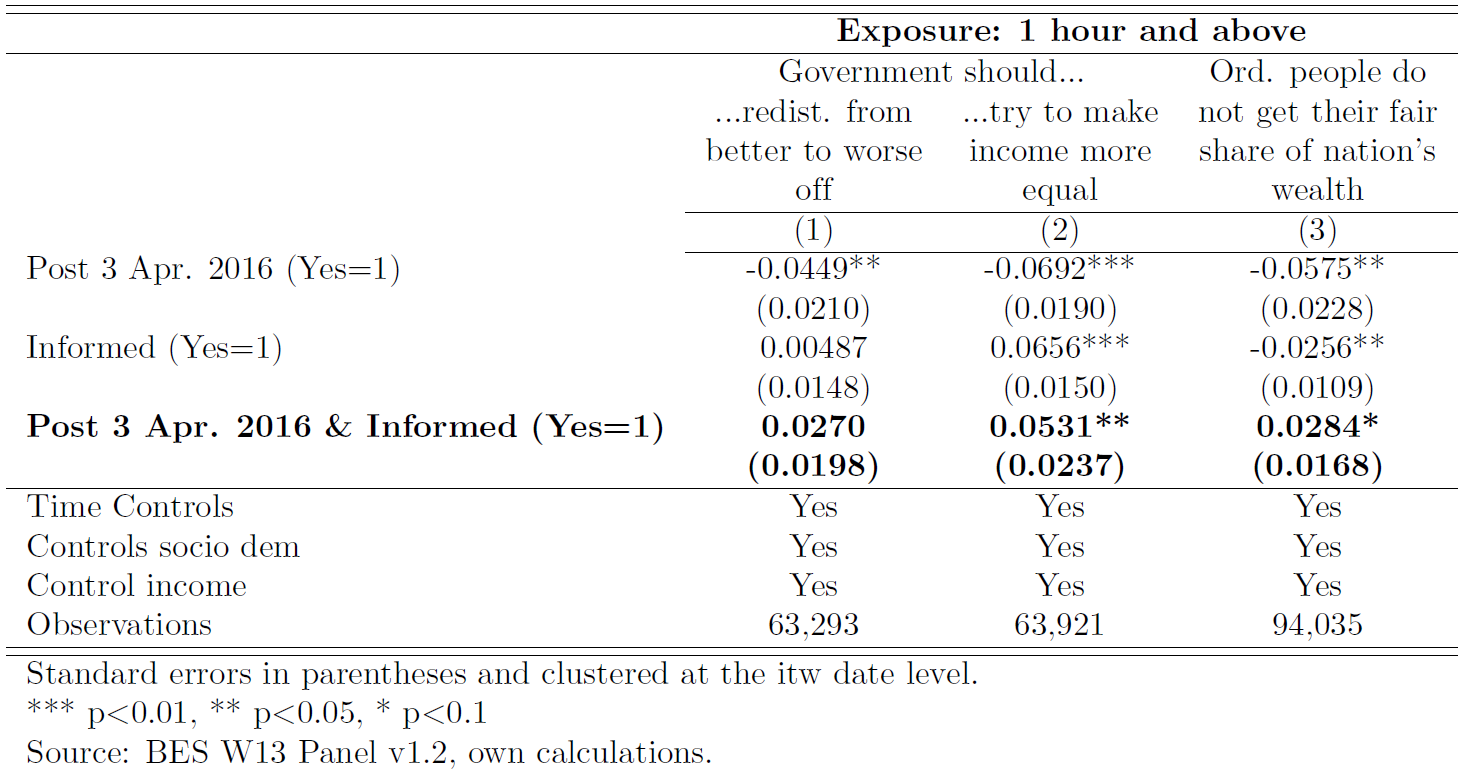

Supplement: S16 Table — (PNG) [file pone.0229394.s023.PNG]

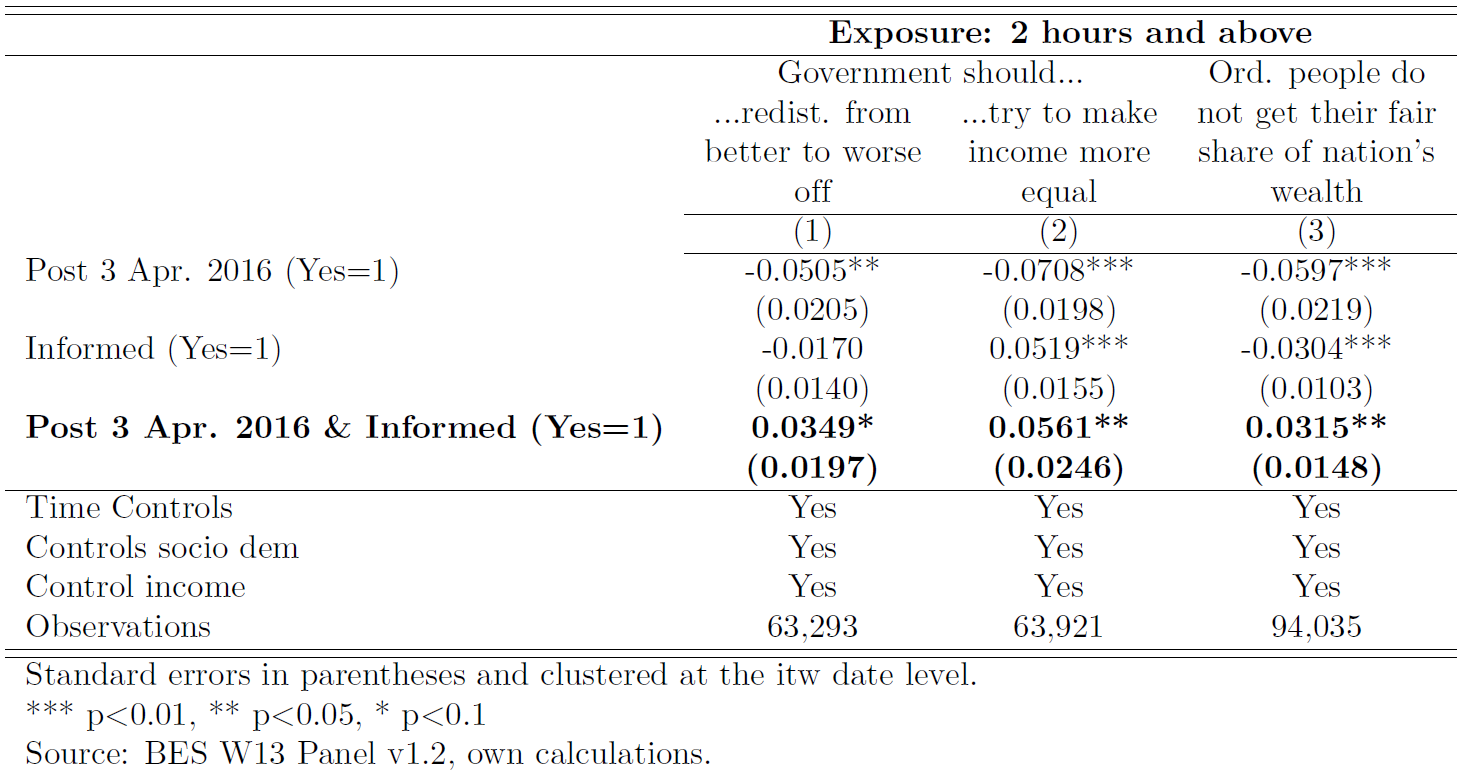

Supplement: S17 Table — (PNG) [file pone.0229394.s024.PNG]

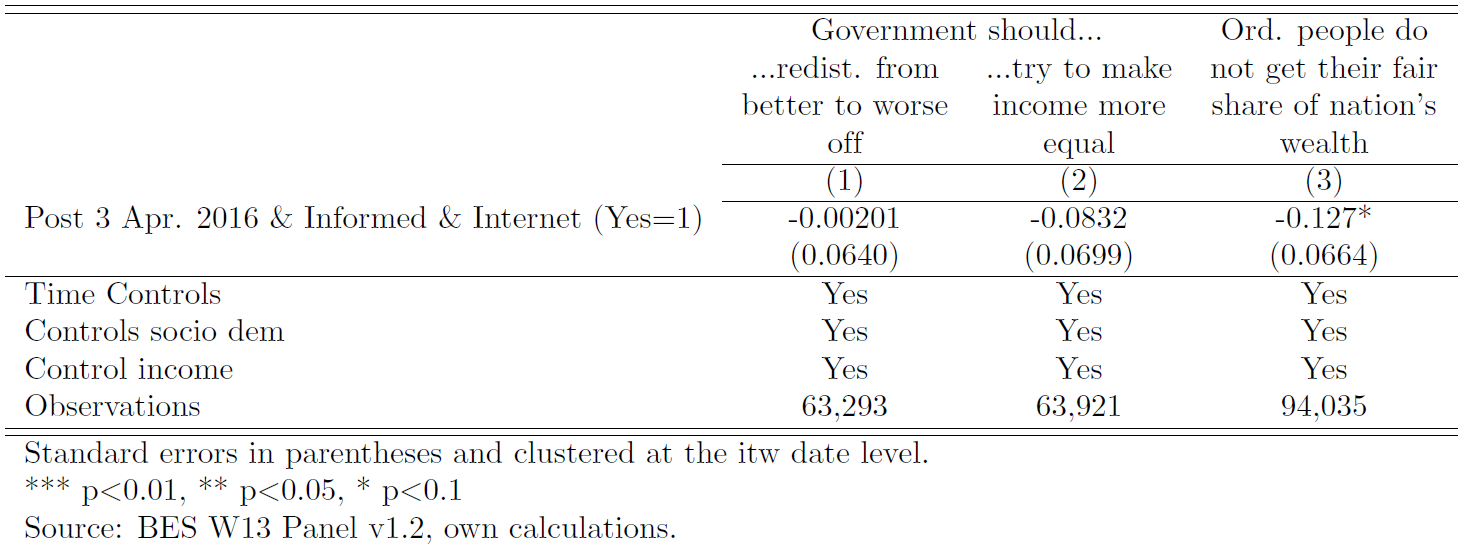

Supplement: S18 Table — (PNG) [file pone.0229394.s025.PNG]

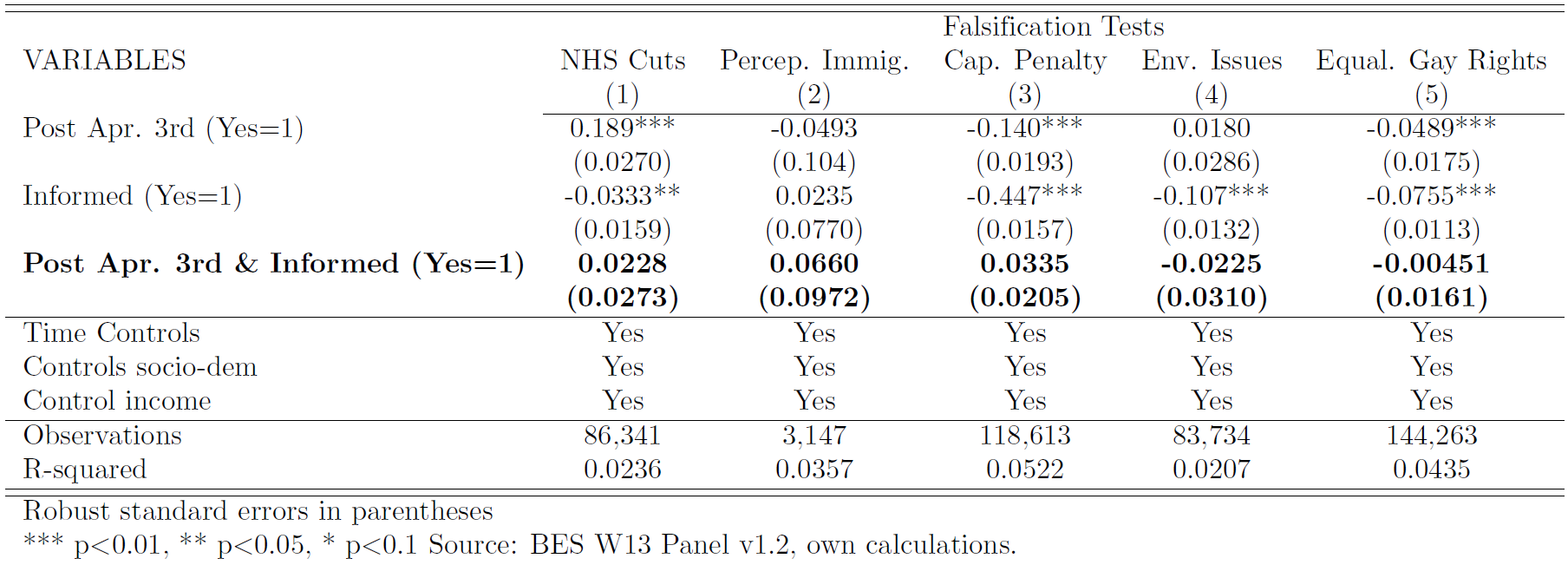

Supplement: S19 Table — (PNG) [file pone.0229394.s026.PNG]

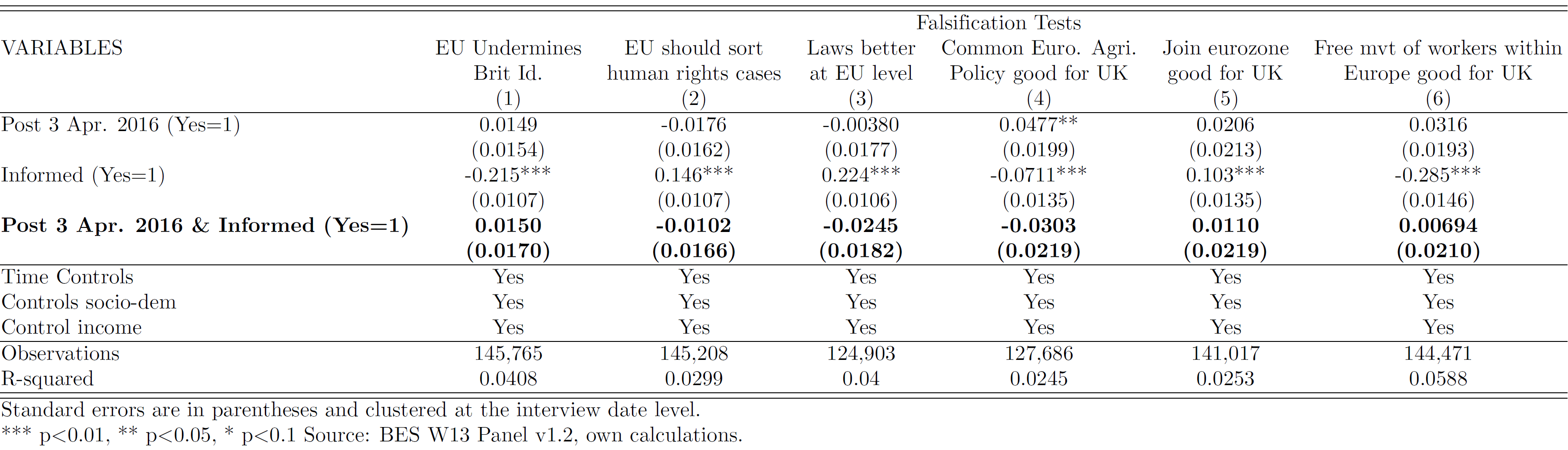

Supplement: S20 Table — (PNG) [file pone.0229394.s027.PNG]

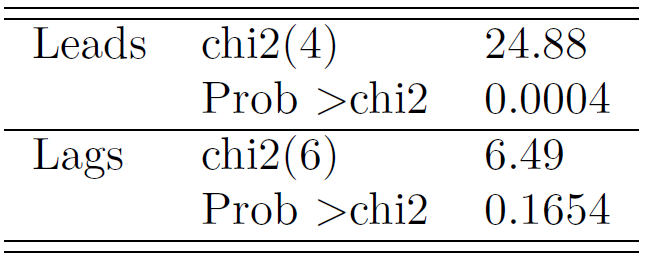

Supplement: S21 Table — (PNG) [file pone.0229394.s028.PNG]

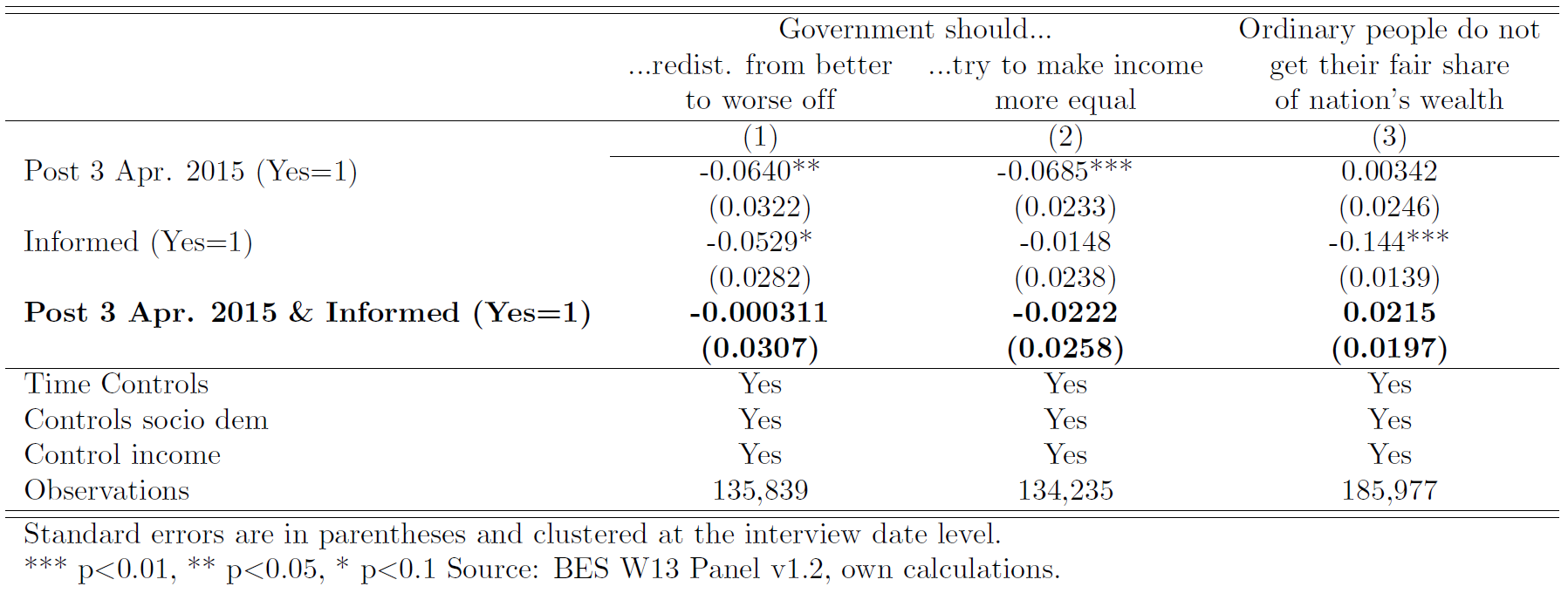

Supplement: S22 Table — (PNG) [file pone.0229394.s029.PNG]

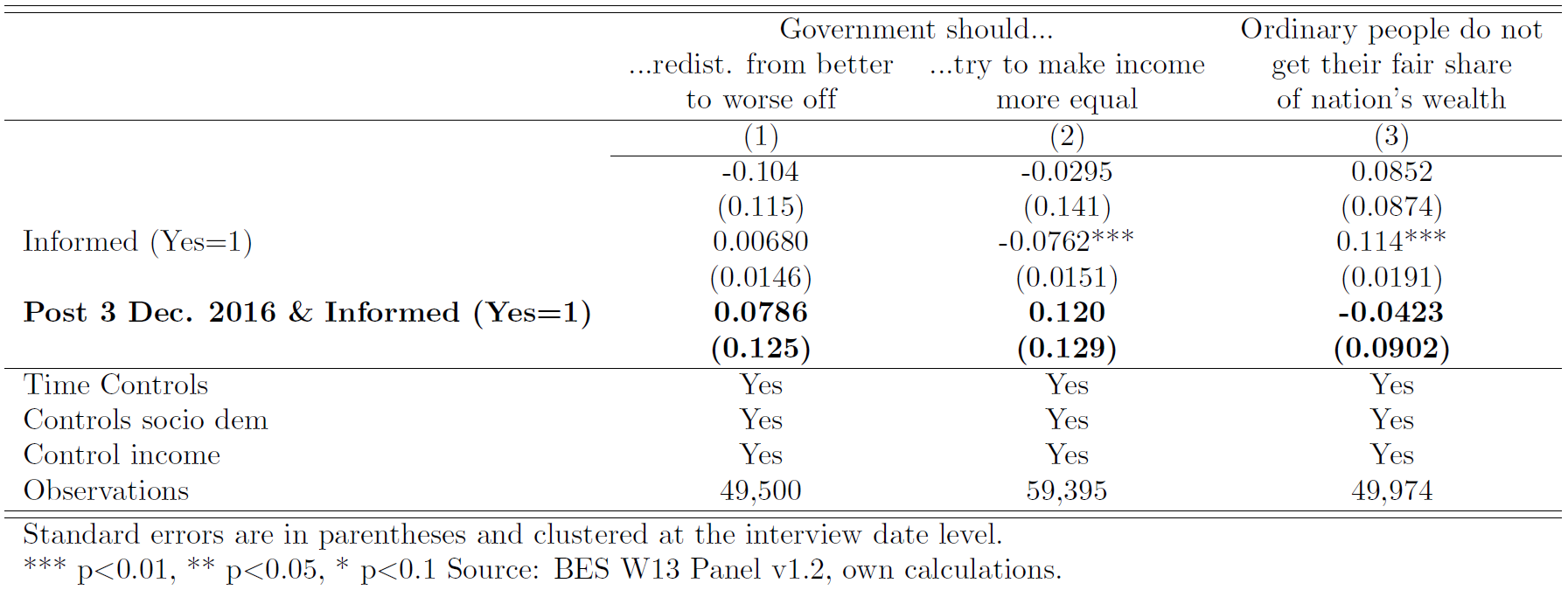

Supplement: S23 Table — (PNG) [file pone.0229394.s030.PNG]

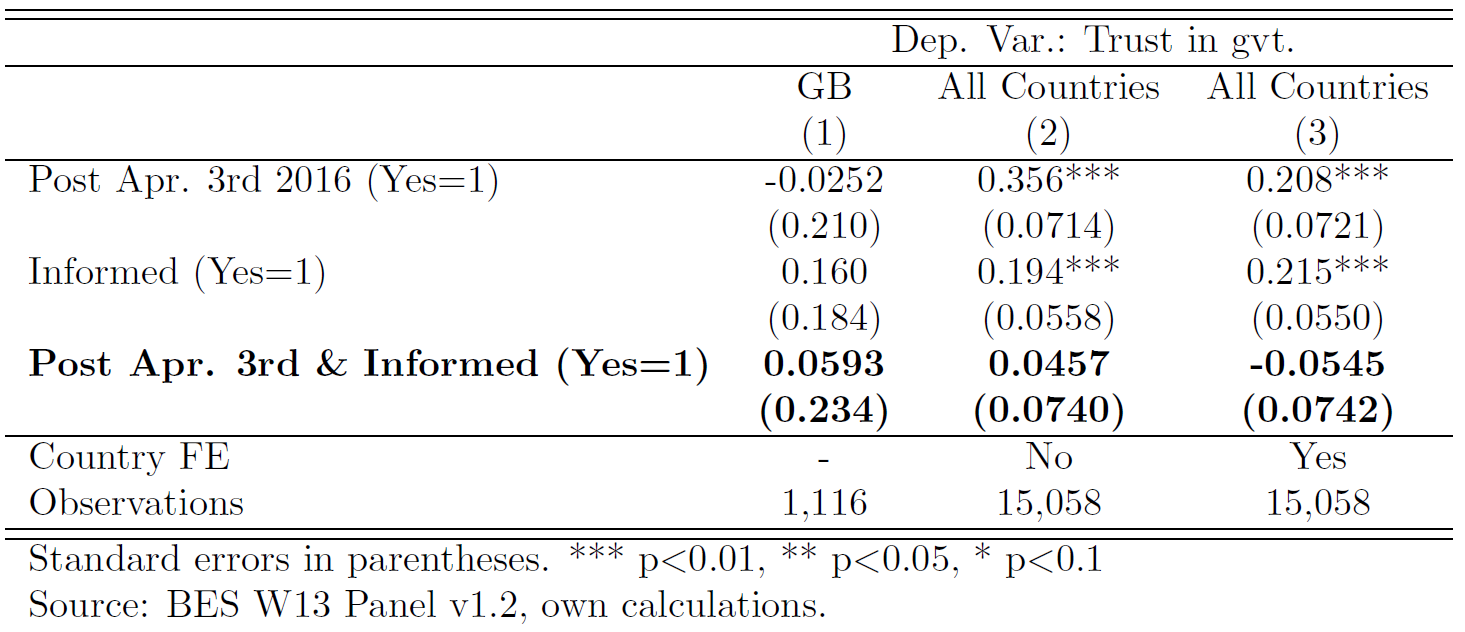

Supplement: S24 Table — (PNG) [file pone.0229394.s031.PNG]

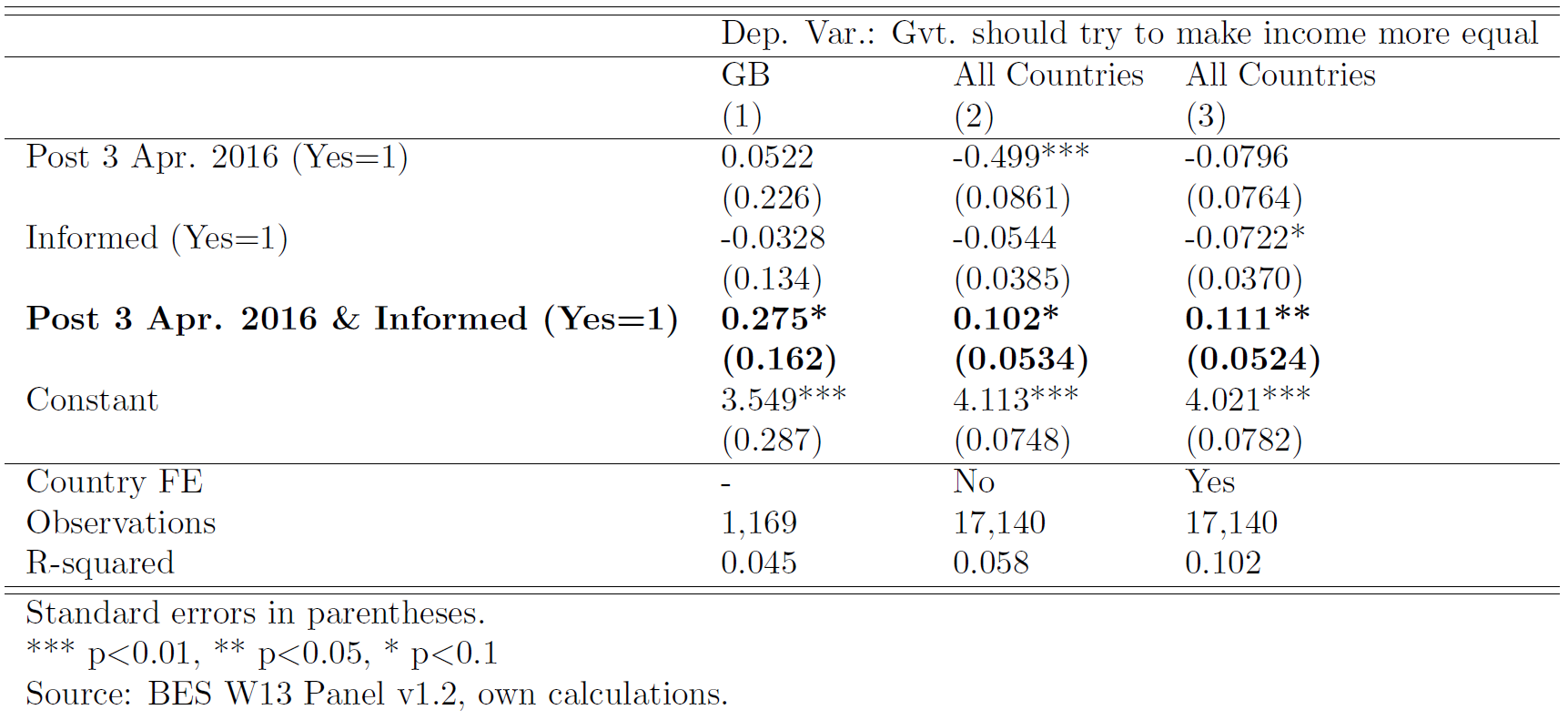

Supplement: S25 Table — (PNG) [file pone.0229394.s032.PNG]

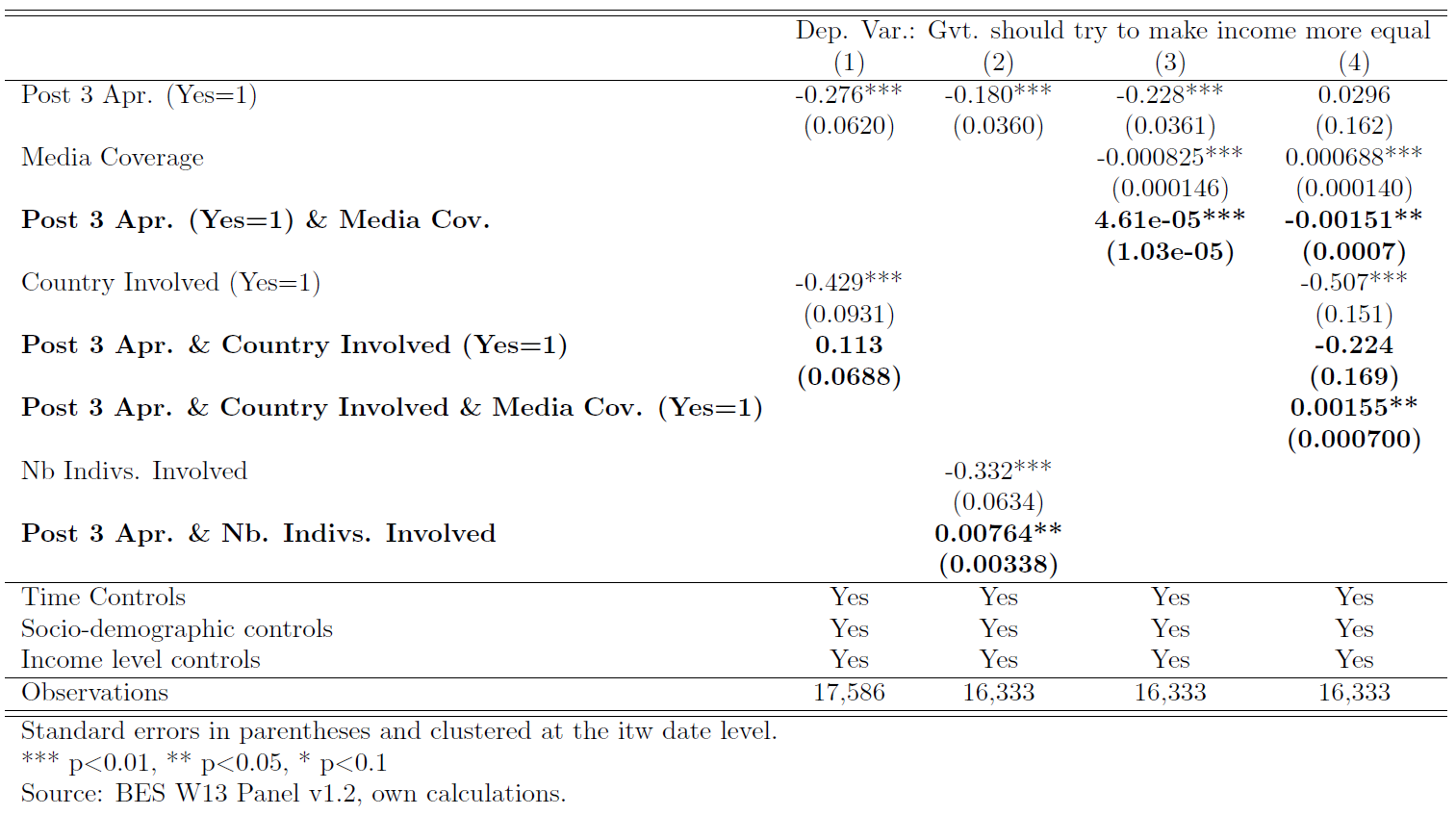

Supplement: S26 Table — (PNG) [file pone.0229394.s033.PNG]
